# Diabetic retinopathy progression in patients under monitoring for treatment or vision loss: external validation and update of a multivariable prediction model

**DOI:** 10.1136/bmjopen-2023-073015

**Published:** 2023-04-03

**Authors:** Sajjad Haider, Nicola Adderley, Mohammad O Tallouzi, Salman Naveed Sadiq, David H Steel, Randhir Chavan, Ijaz Sheikh, Krishnarajah Nirantharakumar, Kym I E Snell

**Affiliations:** 1 Institute of Applied Health Research, University of Birmingham, Birmingham, UK; 2 Birmingham and Midland Eye Centre, Birmingham, UK; 3 Ophthalmology, Royal Victoria Infirmary, Newcastle upon Tyne, UK; 4 Sunderland Eye Infirmary, Sunderland, UK; 5 Newcastle University Biosciences Institute, Newcastle upon Tyne, UK; 6 Sandwell and West Birmingham Hospitals NHS Trust, Birmingham, UK; 7 Eye Department, Surrey and Sussex Healthcare NHS Trust, Redhill, UK; 8 Centre for Prognosis Research, School of Medicine, Keele University, Keele, UK

**Keywords:** Diabetic retinopathy, Medical retina, Ophthalmology, DIABETES & ENDOCRINOLOGY

## Abstract

**Introduction:**

The number of people with diabetes mellitus is increasing globally and consequently so too is diabetic retinopathy (DR). Most patients with diabetes are monitored through the diabetic eye screening programme (DESP) until they have signs of retinopathy and these changes progress, requiring referral into hospital eye services (HES). Here, they continue to be monitored until they require treatment. Due to current pressures on HES, delays can occur, leading to harm. There is a need to triage patients based on their individual risk. At present, patients are stratified according to retinopathy stage alone, yet other risk factors like glycated haemoglobin (HbA1c) may be useful. Therefore, a prediction model that combines multiple prognostic factors to predict progression will be useful for triage in this setting to improve care.

We previously developed a Diabetic Retinopathy Progression model to Treatment or Vision Loss (DRPTVL-UK) using a large primary care database. The aim of the present study is to externally validate the DRPTVL-UK model in a secondary care setting, specifically in a population under care by HES. This study will also provide an opportunity to update the model by considering additional predictors not previously available.

**Methods and analysis:**

We will use a retrospective cohort of 2400 patients with diabetes aged 12 years and over, referred from DESP to the NHS hospital trusts with referable DR between 2013 and 2016, with follow-up information recorded until December 2021.

We will evaluate the external validity of the DRPTVL-UK model using measures of discrimination, calibration and net benefit. In addition, consensus meetings will be held to agree on acceptable risk thresholds for triage within the HES system.

**Ethics and dissemination:**

This study was approved by REC (ref 22/SC/0425, 05/12/2022, Hampshire A Research Ethics Committee). The results of the study will be published in a peer-reviewed journal, presented at clinical conferences.

**Trial Registration number:**

ISRCTN 10956293.

**STRENGTHS AND LIMITATIONS OF THIS STUDY**

- The model will be externally validated in the secondary care setting in which it is intended to be used.
- Extracting the data (not using an existing dataset) ensures the sample size will be large enough for the aims.
- Retrospective cohort design means data can be extracted relatively quickly at minimal cost.
- Opportunity to update the model to include additional predictors, which may improve predictive performance.
- As data are being collected retrospectively, some predictor variables and outcomes may be missing for some patients or trusts. This will be dealt with by using multiple imputation where possible.

## Introduction

**Figure 1:**
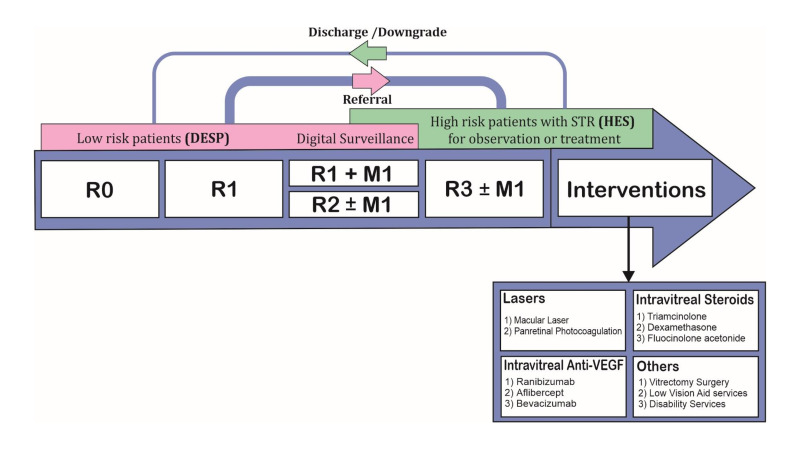
Patient flow diagram (there may be local variations in practices). DESP, Diabetic Eye Screening Programme; HES, hospital eye services; M0, No Maculopathy; M1, Maculopathy present; R0, No retinopathy; R1, Background retinopathy; R2, preproliferative retinopathy; R3, proliferative retinopathy; STR, sight threatening retinopathy; VEGF, vascular endothelial growth factor.

Diabetes mellitus is one of the most common chronic conditions affecting nearly 4.8 million people in UK as of 2019.^1^ With the prevalence rising each year,^2^ there is an ongoing global and UK wide increase in the number of people with diabetes mellitus^3–5^ and consequently diabetic retinopathy (DR). Our study estimated about 1.4 million with any DR and 0.54 million with referable stage DR^2^ in 2017. The detection of DR has also improved through wider population screening, further increasing the demand for hospital eye services (HES).^6^ Diabetes is a major public health concern and uses a significant proportion of the NHS budget, much of which is spent treating the complications of diabetes.^7^ These complications affect blood vessels in the heart, brain, kidney and eyes.^8^ Diabetes is the fourth-leading cause of preventable vision loss in the UK,^9^ and therefore, patients with diabetes are screened regularly for signs of DR. Screening services are organised by the Diabetic Eye Screening Programme (DESP) for patients without DR or with background DR. However, when a patient develops clinical signs of referable retinopathy, including preproliferative DR (R2), proliferative DR (R3) and/or diabetic maculopathy (M1), they are referred to HES or surveillance clinics for closer observation and treatment to prevent vision loss. The patients’ flow within the NHS is depicted in the figure 1.

Most referrals made to HES (50%–78%) will not yet require treatment.^10 11^ Among those that will require treatment, such as patients with diabetic maculopathy, the patients’ condition may be subthreshold (under 400 µm foveal thickness) for treatment and remain so for a period of time. Patients with preproliferative retinopathy are not offered any treatment and are monitored every 3–6 months until they progress to the proliferative retinopathy stage, at which point they receive treatment. Consequent overburdening of HES, combined with under-resourced services may be causing delays in patients being seen and causing harm especially in the higher-risk patients.^12^ Therefore, this bottleneck urgently needs addressing. We propose to mitigate this risk of harm to patients by stratifying patients referred to HES according to their risk of requiring treatment or losing their vision using a clinical prediction model. This would enable higher risk patients to be prioritised and seen sooner.

Clinical prediction models are statistical models that use multiple predictor variables to predict the risk of a clinical outcome.^13^ They can be used by clinicians to stratify care by risk groups based on the predicted probabilities from the model. The DESP uses risk stratification studies to inform suitable screening intervals.^14^ There are also prediction models to identify patients at the highest risk of developing referable DR,^10 15 16^ validated in a UK population.^17^ However, there are currently no such validated prediction models that can be used to stratify care according to risk in patients under the care of HES. Once validated, the Diabetic Retinopathy Progression model to Treatment or Vision Loss (DRPTVL-UK) model could help HES prioritise patients at high risk of vision loss, and to determine suitable follow-up intervals based on an individual’s risk.

The current length of follow-up intervals used within HES is based on the probability of disease progression from a study conducted in the late eighties^18^ and not based on the patient’s individual risk. We therefore aim to predict the progression of DR to treatment stage, to direct resources toward higher-risk patients so that they are followed up and treated before vision failure occurs. We propose that use of a validated risk prediction model will facilitate evidence-based decisions and thus reduce the chance of harm to higher-risk patients.

There are two recent systematic reviews of existing models for predicting the progression of DR among the DESP population.^15 19^ A review by this group of researchers found a total of 14 predictive model development studies of which 11 had been internally validated, 8 had been externally validated and only three without risk of bias.^15^ In a more recent review, there were 16 model development studies for an outcome of referable DR.^20^ Based on these two reviews, it was concluded that none of the existing models were intended for our target population (patients under care of HES/surveillance clinics) and none used the clinically important outcomes of interest (including contemporary treatment modalities and vision loss). Therefore, a prediction model that could be used to identify patients with a higher probability of requiring treatment or at high risk of loss of vision for HES was needed.

In brief, the DRPTVL-UK model was developed in anonymised, retrospective primary care data from IQVIA Medical Research Data (IMRD). From predictors identified in our systematic review,^15^ we selected a set of 19 clinically meaningful candidate predictors of DR progression using the Nominal Group Technique.^21^ A prediction model was then developed considering 15 of the 19 candidate predictors for inclusion based on availability in the dataset.^22^ After variable selection, the final model included seven predictors, namely (1) retinopathy stage, (2) glycated haemoglobin (HbA1c), (3) estimated glomerular filtration rate (eGFR), (4) total serum cholesterol, (5) systolic blood pressure and drug use of (6) insulin or (7) statins. The DRPTVL-UK model demonstrated moderately good discriminative performance (C-statistic=0.74) and very little optimism (0.004) in the internal validation due to the large sample size (13 691 patients).

### Rationale

We now need to assess the model’s predictive performance in a secondary care population to ensure it performs adequately to identify patients at high risk of treatment or vision loss in an HES setting. If this model performs well for predicting risk at time points up to 2 years in the external validation using HES/surveillance clinic data, we propose that it could be used to prioritise individuals at higher risk of vision loss and potentially inform the length of the follow-up intervals after referral to HES/surveillance clinics.

### Objectives and outcome measures/endpoints

The overall aim is to externally validate the multivariable risk prediction model we previously developed, recalibrating to the secondary care population if necessary and updating with additional predictor variables if necessary. The primary objectives are to:

- Assess the external validity of the DRPTVL-UK model for predicting the risk of need for treatment or vision loss up to 2 years after referral in a hospital-based DR population by assessing model calibration, discrimination and net benefit.
- Evaluate whether recalibration of the baseline hazard or linear predictor (combination of predictor effects) improves predictive performance in an HES/surveillance clinic population and whether including additional predictors improves the model’s predictive performance in this population.

Secondary objectives are to:

- Assess the DRPTVL-UK model’s external validity in the subgroup of patients with preproliferative DR (R2) or M1.
- Validate the model across several time points up to 2 years to assess whether it could be used to inform follow-up intervals after referral into HES or a surveillance clinic.

Parallel with the validation, there will be two consensus meetings of expert clinicians and patients to determine how the model can be implemented in practice and establish clinically meaningful thresholds for use.

## Methods and analysis

This prognostic study has been guided by the PROGRESS framework (theme 3 for prognostic models) and will be reported according to the TRIPOD Statement (Transparent Reporting of a multivariable prediction model for individual prognosis or diagnosis).^23^ The study started on 1 July 2022, with data extraction planned to start on 1 April 2023. Study is scheduled to end by the end of December 2023.

### Design and data sources

This retrospective cohort study will use patient data collected from HES/surveillance clinic and other related databases/patient notes from three NHS trusts. Where required information is not available for extraction from the hospitals’ notes, it will be obtained from surveillance clinics if available (and vice versa) by the participating NHS trusts.

### Patients and public involvement

Patients and the public have been involved in the design of this study (see online supplemental appendix 1 for details), and will be involved in the conduct, reporting and dissemination of this research.

10.1136/bmjopen-2023-073015.supp1 Supplementary data

### Study population

The cohort will include type 1 and type 2 patients with diabetes, aged 12 years and over (as patients enter the screening programme from age 12), referred into HES or surveillance clinics with referable DR from DESP for close monitoring and treatment. Data will be collected from three NHS trusts for all patients within the catchment areas of Sandwell and Birmingham, Sunderland or Sussex NHS trusts. Records will be extracted for patients first entering the services between 1 January 2013 and 31 December 2016, with follow-up information extracted up to 31 December 2021. The Birmingham trust cares for an ethnically and socioeconomically diverse range of communities and was chosen to ensure equality, diversity and inclusion. The Sussex trust provides secondary care to a less diverse population and Sunderland is a primarily Caucasian population.

**Figure 2:**
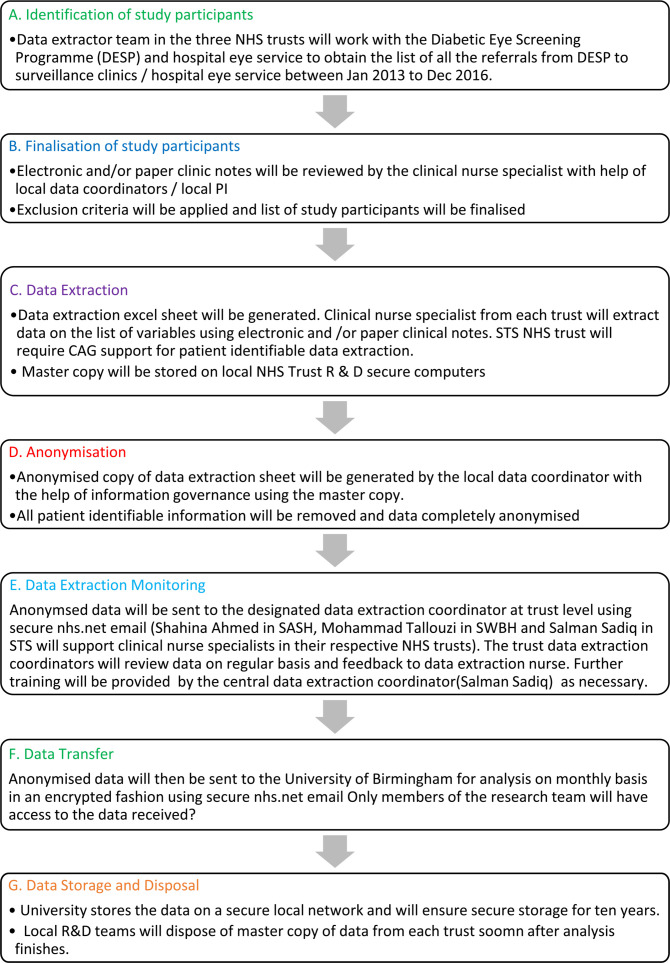
Data flow diagram. CAG, Confidentiality Advisory Group; NHS, National Health Service; PI, Principle investigator; R & D, Research and Development; SASH, Surrey and Sussex Healthcare NHS Trust; STS, South Tyneside and Sunderland NHS trust; SWBH, Sandwell and West Birmingham Hospitals NHS Trust.

Patients with the specific outcome of retinopathy treatment or vision loss at referral or those referred for reasons other than retinopathy will be excluded. Patients objecting to their information being used (through a local or national opt out scheme) will also be excluded. Data flow and management stages are given in figure 2.

### Sample size

A minimum of 200 outcome events are required for external validation using current guidance for survival outcomes.^24^ Every trust receives approximately 200 referrals per year and we expect to have 4 years’ worth of data available for each trust. Therefore, we expect to have approximately 2400 patients from across the three trusts to ensure a minimum of 200 outcomes. Recruitment will stop once the target number is reached.

Using conservative estimates from our development data, we expect 15% of those referred to develop the outcome of interest within 3 years follow-up,^22^ providing at least 360 outcomes in the data that we will collect. For model updating, we will use the method of Riley *et al*^25^ to calculate the minimum sample size required, assuming an event rate of 0.05 per year, mean follow-up of 3.23 years, a default Nagelkerke R^2^ of 0.15 and 19 candidate predictors considered in the model. A minimum of 1810 patients are required with 293 outcome events to target a shrinkage factor of 0.9 ensuring minimal overfitting to the data.

### Data extraction

A data extraction sheet has been prepared using Microsoft Excel and will be piloted prior to use within each trust. Data will be extracted for all predictors at baseline (first HES appointment after referral) and visual acuity level will be collected at each follow-up appointment. Data will continue to be extracted until an outcome of interest occurs, death, lost to follow-up or the end of the study period (31 December 2021).

Personal information such as date of birth (for age calculation), date of diabetes diagnosis and date of death (for censoring or competing risks analysis) will be extracted. However, all the personal identifiable information will be removed after age, diabetes duration and follow-up durations have been calculated.

### Outcomes

The primary outcome for this study is time to first treatment for DR or vision loss. Time will be calculated from referral to HES (baseline is first appointment) until the date of first treatment or vision loss. Time to death will also be recorded and treated as a competing risk if necessary.

### Predictor variables

The previously developed DRPTVL-UK model included seven predictors measured at the time of or close to referral, namely (1) retinopathy stage, (2) HbA1c (mmol/mol), (3) eGFR (mL/min/1.73 m^2^), (4) total serum cholesterol (mmol/L), (5) systolic blood pressure (mm Hg) and drug use of (6) insulin for (7) statins.

Some candidate predictors like the Early Worsening, high non-attendance rate, pregnancy and visual acuity were not previously available in the IMRD database used to develop the model but will be collected from the three trusts. Their definitions are in online supplemental appendices 2 and 3.

### DRPTVL-UK model

We previously developed and internally validated the DRPTVL-UK for the purpose of predicting the risk of vision loss and blindness or need for treatment in patients with referable DR. The model had good discrimination (C-statistic=0.74) and very little optimism in the internal validation. The model^22^ was developed in a primary care population and now needs external validation in the HES/surveillance clinic population, in which it is intended to be used.

The DRPTVL-UK model was developed using Cox regression and later refitted using a flexible parametric approach to obtain the baseline hazard function over time. The model can be used to predict the absolute risk of progression from referable DR to treatment or vision loss occurring within a 2-year period, based on an individual’s risk factor values. Thorough evaluation of the model’s external validity and net benefit is now required to establish whether the model is suitable for use in clinical practice in HES/surveillance clinics.

The DRPTVL-UK model can also be used to predict the time at which an individual reaches a particular risk threshold (to be agreed in a consensus meeting of clinical experts and patients planned after final analysis) which may be useful for determining appropriate follow-up intervals after referral to HES/surveillance clinics. This will be evaluated as a secondary objective.

### External validation of the DRPTVL-UK model

The DRPTVL-UK model will be used to obtain the predicted probability of the outcome over time for every participant within each of the three trusts. Predictive performance of the model will be assessed using measures of discrimination (Harrell’s C-statistic and time-dependent C-statistic), calibration (calibration slope, ratio of observed to expected probabilities, calibration plots at multiple time points up to 2 years) and net benefit using decision curves. Performance measures will be calculated within each hospital and then pooled on an appropriate scale using random effects meta-analysis to account for clustering by hospital.^26^

As another potential use of the model would be to determine appropriate follow-up intervals based on the individual’s risk, it will also be crucial to ensure that the model performs well for predictions at all time points up to 2 years to ensure risk predictions are accurate over time. Therefore, we will also evaluate calibration performance at multiple time points. In addition to this, we will look at the predictions over time (predicted survival curves) and compare these to the observed survival curves for risk groups and other meaningful groupings, for example, DR grade.

In addition to external validation of the model in the whole sample, we will also validate the model within the subgroups of R2/M1 patients to see how well it performs in each.

### Summarising baseline variables

Baseline variables will include predictors measured at referral or shortly before (see table 1 for full list of variables to be collected). Continuous variables will be described using means and SDs (prior to centring and standardisation), binary or categorical variables will be described using frequencies and proportions.

**Table 1:** List of variables for data collection—predictors modified from,^21^ outcomes and competing risk variables

	Group	Required variables	Source, units
1	Ocular features*	Diabetic retinopathy grade	DESP/hospital notes/letter
2		Visual acuity score	Both eyes, log MAR, every visit with date till the outcome
3	Biochemical parameters	Glycated haemoglobin (HbA1c)	From biochemistry database (mmol/mol)
4		Estimated glomerular filtration rate (eGFR)	From biochemistry database, mL/min/1.73 m^2^
5		Total serum cholesterol	From biochemistry database, mmol/l
6	Physical examination	Systolic blood pressure	From nursing notes, mm Hg
7	Diabetes treatment	Statin	From General Practitioner (GP) letter/diabetology notes
8		Insulin	From GP letter/diabetology notes
9	NGT*	Pregnancy	During the preceding 2 years before referral
10		Early worsening	To be calculated at baseline (details in online supplemental appendices 2 and 3).
11		Frequent DNA/cancellations (total, two consecutive sets)	With dates (patient administration system)
12	Competing risk	Date of death if occurring before the treatment/ vision failure/date of discharge	Patient administration system, deidentify after
13	Outcome/follow-up	Date of treatment (first ever)/vision failure/ discharge/transfer/end of the study—end December 2021 (whichever happens first)	Patient notes
14	Demographics	Age, gender, ethnicity and deprivation score	Patient notes

*Among the predictors, ocular features (DR stage in each eye) and visual acuity will be recorded for both eyes at every visit along with the date of measurement. For analysis, the eye with the higher DR grade will be used (R3M1>R3>R2 M1>M1>R2). For other predictors in the model, values up to and including the first referral (baseline) appointment will be used, provided they occur in the 12 months prior to the referral appointment. The measurement closest to the referral (baseline) appointment will be used. Please also see online supplemental appendices 2 and 3 for details.

DESP, Diabetic Eye Screening Programme; DNA, did not attend; DR, diabetic retinopathy; NGT, Nominal Group Technique.

### Model recalibration and updating

If necessary, we will recalibrate the model to the HES population (eg, by updating the baseline survival function or recalibrating the linear predictor).^27^ In addition, we will investigate whether updating the model to include additional predictors that were not available in the development dataset improves the predictive performance. Visual acuity, early worsening, pregnancy and frequent ‘did not attend’, were identified as candidate predictors based on expert opinion and evidence evaluation.^21^ To update the model with additional predictors, flexible parametric models (Royston-Parmar models) will be fitted using a multivariable fractional polynomial approach to consider non-linear functions for continuous variables while using backward elimination for the additional predictors considered.^28^ We will use a p>0.157 as a proxy for selection based on Akaike information criterion.^29^ All predictors from the original model will be forced to remain in the model regardless of statistical significance, therefore, only the four additional variables will be tested. The predictive performance of the updated model will be evaluated using internal–external cross-validation^30^ in which the model is developed using the data from two hospitals and externally validated in the third. This is then repeated a total of three times, each time reserving a different hospital for external validation. Predictive performance will be evaluated in each ‘external’ hospital using the same measures of discrimination and calibration as previously described and will be summarised across the hospitals using random-effects meta-analysis. Predictive performance of the updated model will be compared with the original model.

### Clinical benefit

We will also evaluate the clinical benefit of the model using decision curve analysis in which the net benefit of using the model at different threshold probabilities is plotted and compared with strategies of intervention for all (following up everyone more frequently) or intervention for no-one (no-one followed up more frequently).^31^

### Missing data

Missing data is a common problem in clinical data and needs to be appropriately accounted for in analyses. An audit using hospital notes from Sunderland Eye Infirmary showed physical examination variables of systolic and diastolic blood pressure nearest to referral were recorded in the clinical notes of 72% of patients; biochemical variables of HbA1c were recorded for 83% of patients, eGFR and cholesterol in 95.5% of patients, measured near to referral. In each case, we will consider why the values might be missing to understand whether a missing at random assumption is reasonable or whether missingness is likely to be informative. For variables missing for <40% of patients, missing data will be handled by multiple imputation using chained equations assuming data are missing at random. The missing at random assumption is an untestable one but data checks comparing characteristics of patients with missing values to those without will be performed to assess if there are any obvious problems with the assumption. To preserve any clustering that may be present, data will be imputed for each hospital separately. The imputation model will include all predictors as well as the outcome using the event indicator and estimate of the cumulative hazard function. Auxiliary variables will be considered to improve the missing at random assumption. The number of imputed datasets will be set at least equal to the percentage of observations of missing data for any of the variables of interest.^32^

### Clinical consensus

Alongside the statistical analysis, clinical consensus workshops will take place to help determine clinically meaningful threshold probabilities for net benefit analysis and for use of the model in practice. This will include discussion and agreement on a suitable threshold for identifying higher risk patients and potential thresholds for determining the follow-up intervals, prior to decision curve analysis. After the results of the external validation including the decision curve analysis are available, they will be presented to the ophthalmic expert committee panel for discussion on how the model can be implemented.

The consensus process was first used in the USA in the early 1970s to address the National Institutes of Health development programme to seek agreement on the safety and efficacy of medical procedures, drugs and devices.^33^ Consensus development meetings were introduced to the UK health system to discuss healthcare policies and its implementation in clinical practice.^34^

The consensus process will be used in this expert group to reduce the range of potential options presented to facilitate joint decision making by the group on the most appropriate choice of the model implementation strategies. The consensus process will help us evaluate the list of options and combine them if an overlap is noted between different options. It can also accommodate the inclusion of further options, check for redundancy between included options and reach agreement through sharing information and knowledge of the participants.^35^ The consensus process described below also enhances the critical thinking of the key stakeholders and facilitates joint decision making of the diverse groups.^36^ Communication and cooperation between participants are key to reach successful agreement on the options discussed and to increase the chances of wider acceptance for implementation.^37^ Here, we aim to reach an agreement on participants’ opinions on the various options under consideration.

In this study, participants will be asked to rate the importance of each of the options based on a nine-point Likert scale that has been adopted in the COMET consensus style; (1–3=less important, 4–6=important and 7–9=critical) using a 70% threshold agreement to score the quality of evidence for outcomes in systematic reviews, and has been adopted in other core outcome development research groups using Delphi methods.^38^ Therefore, participants will be asked to vote on whether particular risk groupings should be considered for the final DRPTVL-UK model, excluded or require further discussion and refinement. For each option presented, the proportion of participants scoring 1–3, 4–6 and 7–9 on the nine-point Likert scale will be calculated for each item. ‘Consensus in’ will be defined as greater than 70% of participants scoring as 7–9. ‘Consensus out’ is based on an item being scored 1–3 by more than 70%. No consensus is based on an item where the level of importance was not decided due to uncertainty.^39^ We anticipate that the group joining the consensus process will include 10–15 participants, ensuring an appropriate balance of representation of the different stake holder groups.

### Software

All statistical analyses will be done using Stata V.16 and R V.4.1 or later versions.

### Ethics and dissemination

For the external validation, routine practice retrospective data including the lists of all NHS numbers of referrals for the years 2013–2016 will be kept on NHS trusts’ R & D secure computers in lockable rooms. Population characteristics like age at baseline/date of birth for age range, date of death for competing risk analysis will be extracted, but after calculation of age, gender distribution and lengths of follow-up durations, all the personal identifiable information will be removed to protect the patient privacy/confidentiality. Data extractors will be clinical nurse specialists employed by the NHS trusts’ R & D. No sensitive data will be extracted. Identifiable data will be removed from data extracted and only anonymised data will be sent to University of Birmingham (UoB) in encrypted fashion. This anonymised data will be stored on secure UoB computers with appropriate access controls, in lockable rooms.

Personal identifiable data will be deleted using deletion software at the time of study end by the contributing NHS trusts. Small number suppression (≤7, as per NHS Digital practice for Hospital Episode Statistics Admitted Patient Care data) will apply.^40^

For the consensus process, write ups will not mention any direct quotes, the NHS trust or any individual expert’s identity. This anonymised data will be stored on secure NHS/UoB computers for 10 years to allow for all possible publications.

The plans for dissemination include peer reviewed publication, presentation to professional/PPIE bodies and development of an electronic calculator application to allow risk-based prioritisation of their follow-up, after direct entry of clinical information. However, further research may be required to assess the clinical and economic impact of the final model. Social media will also be used to disseminate findings.

## Limitations

Missing data is the main limitation foreseen. Small sample size for subgroup analysis may reduce the model’s discrimination ability.

## Supplementary Material

Reviewer comments

Author's manuscript
