# Supplementary material for: Diabetic retinopathy progression in patients under monitoring for treatment or vision loss: external validation and update of a multivariable prediction model
Source: BMJ Open. 2023 Apr 3;13(4):e073015. doi: 10.1136/bmjopen-2023-073015 (PMC10083856; doi:10.1136/bmjopen-2023-073015)
Supplement: Supplementary data [file bmjopen-2023-073015supp001.pdf]

DRPTVL-UK Model validation

Appendices

Appendix 1: Pre-application patients and public involvement and engagement (PPIE) and later consultation with stake holders

The author has previously published on the predictors for progression of DR according to the James Lind Alliance priority setting (1) (priority 3 under retinal vascular disease/sight loss and vision) (2). The present study addresses priority 8 on the same top 10 research priorities (barriers that prevent diabetic patients having regular eye checks). For wider clinical expert input, we held consultation meetings with ophthalmologist colleagues with DR as their special interest, their DESP colleagues, diabetologists interested in DR and GP's with specialist interest in diabetes. They all provided detailed written feedback which was incorporated into the research design for funding application. The table below summarises the PPIE activities undertaken.

Table : List of PPIE activities (mostly pre-application)

Supplementary File

DRPTVL-UK Model validation

**PPIE Activity Report -** Modified from GRIPP 2 (3)		
1	**Aims**	The aim of this PPIE exercise was to get patients' perspective about this research and to involve them in the design of the study and in the grant application.
2	**Objectives**	To recruit a diverse group for equitable representation.
		To train patients with diabetic retinopathy under care of the hospital eye services joining the group in PPIE.
		To ensure the use of friendly and plain language in the lay summary.
		To get PPIE input into the research project.
		To form a patient steering group and to recruit a patient advocate as a co- applicant with a deputy.
3	**Methods**	Recruitment through Diabetes UK, Clinical Research Network, three NHS trusts (northeast, midlands and southeast), local research networks, and through GP forums in order to include a diverse group and to ensure equitable access. The patients had been living with diabetic retinopathy and had been under the care of hospital eye services for at least one year.
		A presentation on all aspects of the research followed by questions and answers followed by open ended discussion
		Requested a volunteer to help write the plain English summary.
		In the presentation, we explained important themes of the research design and plans, but also ensured an adequate open-ended discussion to cover unforeseen patient perspectives, experiences, and concerns. We then brainstormed to gain further patient input.
		We invited two volunteers to act as co-applicants as patient advocates.
4	**Study results**	The patient advisory meeting was held remotely on 4th March 2021. 8 participants (including a GP representative) from three different regions of various ages and of different ethnicities attended. Patients without any internet access were invited into a GP practice to provide access to the virtual meeting.

Supplementary File

DRPTVL-UK Model validation

		Participants reviewed the presentation, asked questions, engaged in discussion, and responded to the meeting minutes. They were supportive of the research and felt it will be beneficial for patients.
		One patient revised the summary to make it easier to read through user-friendly language.
		There were two important comments from patients on study design. They wanted to ensure safety for the patients where model does not accurately predict and did not want the ceiling for follow-up intervals to be as high as 2 years as in Diabetic Eye Screening Programme.
		Two volunteers accepted the invitation to act as patient advocates, one as co-applicant and the other as deputy. The group also agreed to be part of patient steering group and play a key role in disseminating the results of the study to the public, patients, their families, and carers.
5	**Discussion and conclusions**	Patients' perspectives regarding the follow up intervals, to be designed up to a maximum of 2 years, was taken on board. The risk arising from uncertainty in the model predictions will be mitigated by raising this issue in the consensus meeting for further discussion before finalising outputs.
6	**Reflections/critical perspective**	A PPIE group comprising a relevant population is now in place. This needs to grow in size for sustainability.

PPIE activity report (Modified from GRIPP 2, Short form https://doi.org/10.1136/bmj.j3453)

After receiving the feedback from NIHR panel on the stage 1 application, we discussed various comments with an ophthalmic expert panel. We have incorporated their advice into this protocol, added a secondary objectives section and added further analysis to external validation in the methods section.

Supplementary File

DRPTVL-UK Model validation

Before starting the data permission applications and regulatory approvals, we tested the acceptability of using anonymised patient data in this study without consent by sending an e mail to the group asking this question. Following were the responses received.

1) "I can't foresee any issues with using patient data so long as it has been completely anonymised".
2) "I am happy for mine to be used".
3) "I think it's a great idea and helps with the project".

Appendix 2: Feasibility of NGT suggested variables:

Early worsening of DR (EWDR)

EWDR arises within 6 months after abrupt improvement of glucose control (> 4% or > 20.2 mmol/mol, during intensive treatment - insulin pump therapy and after pancreas transplantation or bariatric surgery). Follow up is required over the following 12 months. EWDR is often transient, with regression of retinal signs after 12 months in the Oslo study in all except four patients [8] and in nearly half of the DCCT patients (4). We shall extract HBA1c twice before baseline.

Audit data from a trust contributing data, impression was that this variable is not well recorded. From prediction point of view, we can look at feasibility of it once data is available. If feasible, then can include it in the model to see if it makes a difference to the model performance. For the patients with the outcomes of treatment, we shall look back at the last 12/12 of HBA1c levels and the evidence of intensive treatment, bariatric / pancreatic surgery.

Pregnancy: While pregnancy is associated with progression of diabetic retinopathy (5), and in type 1, it induces a transient increase (2.5-fold) in the risk of retinopathy (6). There is also a low risk of progression of DR in type 2 diabetes (7) as well. Increased ophthalmic surveillance is needed during pregnancy and the first year postpartum. From modelling perspective this variable may not be relevant as most patients are beyond reproductive age (> 60 years mean). But we shall use the variable as history of pregnancy less than two years before the outcome of need for treatment.

Supplementary File

DRPTVL-UK Model validation

Frequent DNA / Cancellations

Patients with history of non-attendance of diabetic eye screening for two consecutive years are at increased risk of developing Sight Threatening Diabetic Retinopathy (8). Evidence of this in patients with referral retinopathy under care of surveillance clinic or hospital services does not exist. However this was voted 8th out of 33 predictors in a nominal group technique exercise (2) attended by ophthalmic clinicians. We shall use this variable during the external update of the model. We shall collect data on total number of non-attendance and cancellations and no of > 1 consecutive non-attendance or cancellations before the baseline (first HES appointment records).

Appendix 3: Important definitions

- Early worsening: “DR progression to treatment requiring stage during the first year after rapid improvement in blood glucose will be considered EWDR” if there is history of intensive treatment / bariatric / pancreatic surgery (4).
- Follow up: From the first appointment after referral by DESP to first treatment (laser / injection) or vision failure, whichever comes first, death, discharge, transfer or end of the study.
- Outcome: This is a composite of treatment (photocoagulation, injection, vitrectomy) or vision failure (vision loss or blindness)
- Treatment: photocoagulation, Intraocular injection treatment with any anti VEGF or steroid injections laser or vitreous surgery
- Vision failure: Loss of three lines of vision (10 to 15 letters on EDTRS) or more, only if it happens before treatment. (Conversions-Between-Letter-LogMAR-and-Snellen-Visual-Acuity-Scores.png (605×725) (researchgate.net), (VALIDITY OF OUTCOME MEASURES - Ranibizumab (Lucentis) - NCBI Bookshelf (nih.gov)

Supplementary File

DRPTVL-UK Model validation

References

1. James Lind Alliance. Priority Setting Partnerships, Retinal Vascular Disease Top 10 [cited 2013. Available from: https://www.jla.nihr.ac.uk/priority-setting-partnerships/sight-loss-and-vision/top-10-priorities/retinal-vascular-disease-top-10.htm.
2. Haider S, Sadiq SN, Lufumpa E, Sihre H, Tallouzi M, Moore DJ, et al. Predictors for diabetic retinopathy progression—findings from nominal group technique and Evidence review. BMJ Open Ophthalmology. 2020;5(1):e000579.
3. Staniszewska S, Brett J, Simera I, Seers K, Mockford C, Goodlad S, et al. GRIPP2 reporting checklists: tools to improve reporting of patient and public involvement in research. BMJ. 2017;358:j3453.
4. Feldman-Billard S, Larger E, Massin P. Early worsening of diabetic retinopathy after rapid improvement of blood glucose control in patients with diabetes. Diabetes Metab. 2018;44(1):4-14.
5. Klein BE, Moss SE, Klein R. Effect of pregnancy on progression of diabetic retinopathy. Diabetes care. 1990;13(1):34-40.
6. DCCT. Effect of pregnancy on microvascular complications in the diabetes control and complications trial. The Diabetes Control and Complications Trial Research Group. Diabetes care. 2000;23(8):1084-91.
7. Rasmussen KL, Laugesen CS, Ringholm L, Vestgaard M, Damm P, Mathiesen ER. Progression of diabetic retinopathy during pregnancy in women with type 2 diabetes. Diabetologia. 2010;53(6):1076-83.
8. Forster AS, Forbes A, Dodhia H, Connor C, Du Chemin A, Sivaprasad S, et al. Non-attendance at diabetic eye screening and risk of sight-threatening diabetic retinopathy: a population-based cohort study. Diabetologia. 2013;56(10):2187-93.

Supplementary File
