# Supplementary material for: Diabetic retinopathy progression in patients under monitoring for treatment or vision loss: external validation and update of a multivariable prediction model
Source: BMJ Open. 2023 Apr 3;13(4):e073015. doi: 10.1136/bmjopen-2023-073015 (PMC10083856; doi:10.1136/bmjopen-2023-073015)
Supplement: Author's manuscript [file bmjopen-2023-073015.draft_revisions.pdf]

BMJ Open is committed to open peer review. As part of this commitment we make the peer review history of every article we publish publicly available.

When an article is published we post the peer reviewers' comments and the authors' responses online. We also post the versions of the paper that were used during peer review. These are the versions that the peer review comments apply to.

The versions of the paper that follow are the versions that were submitted during the peer review process. They are not the versions of record or the final published versions. They should not be cited or distributed as the published version of this manuscript.

BMJ Open is an open access journal and the full, final, typeset and author-corrected version of record of the manuscript is available on our site with no access controls, subscription charges or pay-per-view fees (http://bmjopen.bmj.com).

If you have any questions on BMJ Open's open peer review process please email info.bmjopen@bmj.com

Diabetic Retinopathy Progression in patients under monitoring for treatment or vision loss: External Validation, and update of a Multivariable Prediction Model.

Journal:	*BMJ Open*
Manuscript ID	bmjopen-2023-073015
Article Type:	Protocol
Date Submitted by the Author:	21-Feb-2023

Complete List of Authors:	Haider, Sajjad; University of Birmingham, Institute of Applied Health Research<br>Adderley, Nicola; University of Birmingham, Institute of Applied Health Research<br>Tallouzi, Mohammad O.; Birmingham and Midland Eye Centre<br>Sadiq, Salman ; Royal Victoria Infirmary, Ophthalmology; Sunderland Eye Infirmary,<br>Steel, David; Sunderland Eye Infirmary; Newcastle University Biosciences Institute<br>Chavan, Randhir; Sandwell and West Birmingham Hospitals NHS Trust<br>Sheikh, Ijaz; Surrey and Sussex Healthcare NHS Trust<br>Nirantharakumar, Krishnarajah; University of Birmingham, Institute of Applied Health Research<br>Snell, Kym IE; Keele University, Centre for Prognosis Research, School of Medicine, Keele University

Keywords:	Diabetic retinopathy < DIABETES & ENDOCRINOLOGY, Medical retina < OPHTHALMOLOGY, Ophthalmology < SURGERY

SCHOLARONE™
Manuscripts

DRPTVL-UK Model validation

Diabetic Retinopathy Progression in patients under monitoring for treatment or vision loss: External Validation, and update of a Multivariable Prediction Model.

Sajjad Haider[1], Nicola J. Adderley[1], Mohammad O. Tallouzi[1,2] Salman Naveed Sadiq[3], David H. Steel[3,4], Randhir Chavan[2], Ijaz Sheikh[5], Krishnarajah Nirantharakumar[1,] Kym I. E. Snell[6]

Corresponding author:

Sajjad Haider, Institute of Applied Health Research, University of Birmingham, Birmingham, United Kingdom

E mail: S.Haider.2@bham.ac.uk

Acknowledgments

We would like to acknowledge the contributions of the expert ophthalmic committee that provided valuable advice and feedback leading to this project being funded. Thanks go to Shahina Ahmed (Surrey and Sussex Hospitals NHS Trust, Redhill, UK), Komala Vedivelue (Durham and Darlington Foundation Trust, UK), Ticiana Criddle (Liverpool University Hospitals Foundation Trust, UK), Maged Habib (South Tyneside & Sunderland NHS Foundation Trust, Honorary Clinical Senior Lecturer. Biosciences Institute. Newcastle University, UK), Daniella vaideanu-Collins (James Cook University Hospital, Marton Road, Middlesbrough, UK), Ramu Muniraju (Surrey Diabetic Eye screening programme, UK), Bob Ryder (Sandwell and West Birmingham Hospitals NHS Trust, Birmingham, UK), Ben Field (Faculty of Health & Medical Sciences, University of Surrey, and Department of Diabetes & Endocrinology, Surrey & Sussex Healthcare NHS Trust UK), Ramesh Sivaraj (University Hospital Birmingham Foundation Trust, UK), Khasheen Alam (Burbury Medical centre Birmingham, UK), Manir Aslam (Broadway Health Centre Birmingham UK), Christopher Sainsbury (Applied Health Research University of Birmingham, UK).

We also like to express our gratitude to the expert patients' panel for their involvement and valuable inputs during pre-funding application process (during research design) and during ethics / CAG application for approval.

Main Text Document

For peer review only

DRPTVL-UK Model validation

ABSTRACT

Introduction

The number of people with diabetes mellitus is increasing globally and consequently so too is diabetic retinopathy (DR). Most diabetic patients are monitored through the diabetic eye screening programme (DESP) until they have signs of retinopathy and these changes progress, requiring referral into hospital eye services (HES). Here they continue to be monitored until they require treatment. Due to current pressures on HES, delays can occur, leading to harm. There is a need to triage patients based on their individual risk. At present, patients are stratified according to retinopathy stage alone, yet other risk factors like HBA1c may be useful. Therefore, a prediction model that combines multiple prognostic factors to predict progression will be useful for triage in this setting to improve care.

We previously developed a Diabetic Retinopathy Progression model to Treatment or Vision Loss (DRPTVL-UK) using a large primary care database. The aim of the present study is to externally validate the DRPTVL-UK model in a secondary care setting, specifically in a population under care by HES. This study will also provide an opportunity to update the model by considering additional predictors not previously available.

Methods and analysis

We will use a retrospective cohort of 2400 diabetic patients aged 12 years and over, referred from DESP to the NHS hospital trusts with referable DR between 2013 and 2016, with follow-up information recorded until December 2021.
We will evaluate the external validity of the DRPTVL-UK model using measures of discrimination, calibration and net benefit. Additionally, consensus meetings will be held to agree on acceptable risk thresholds for triage within the HES system.

Ethics and Dissemination
This study was approved by

- Ethical approval for the study has been granted by the National Research Ethics Service (NRES) West Midlands – Hampshire Research Ethics Committee (Reference number 22/SC/0425),
- CAG reference: 22/CAG/0149
- Registration: ISRCTN 10956293

Main Text Document

For peer review only - http://bmjopen.bmj.com/site/about/guidelines.xhtml

DRPTVL-UK Model validation

The results of the study will be published in a peer-reviewed journal and presented at clinical conferences.

Strengths and limitations of this study

- The model will be externally validated in the secondary care setting in which it is intended to be used.
- Extracting the data (not using an existing dataset) ensures the sample size will be large enough for the aims.
- Retrospective cohort design means data can be extracted relatively quickly at minimal cost.
- Opportunity to update the model to include additional predictors which may improve predictive performance.
- As data are being collected retrospectively, some predictor variables and outcomes may be missing for some patients or trusts. This will be dealt with by using multiple imputation where possible.

INTRODUCTION

Diabetes mellitus is one of the most common chronic conditions affecting nearly 4.8 million people in UK as of 2019 (1). With the prevalence rising each year (2), there is an ongoing global and UK wide increase in the number of people with diabetes mellitus (3-5) and consequently diabetic retinopathy DR. Our study estimated about 1.4 million with any DR and 0.54 million with referrable stage DR (2) in 2017. The detection of DR has also improved through wider population screening, further increasing the demand for HES (6). Diabetes is a major public health concern and uses a significant proportion of the NHS budget, much of which is spent treating the complications of diabetes (7). These complications affect blood vessels in the heart, brain, kidney and eyes (8). Diabetes is the fourth leading cause of preventable vision loss in the UK (9), and therefore patients with diabetes are screened regularly for signs of DR. Screening services are organised by the Diabetic Eye Screening Programme (DESP) for patients without DR or with background DR. However, when a patient develops clinical signs of referable retinopathy, including pre-proliferative diabetic retinopathy (R2), proliferative diabetic retinopathy (R3) and / or diabetic maculopathy (M1), they are referred to HES or surveillance clinics for closer observation and treatment to prevent vision loss. The patients' flow within the NHS is depicted in the figure 1.

Most referrals made to HES (50 to 78%) will not yet require treatment (10, 11). Among those

For peer review only - http://bmjopen.bmj.com/site/about/guidelines.xhtml

that will require treatment, such as patients with diabetic maculopathy, the patients' condition may be subthreshold (under 400 microns foveal thickness) for treatment and remain so for a period of time. Patients with pre-proliferative retinopathy are not offered any treatment and are monitored every three to six months until they progress to the proliferative retinopathy stage, at which point they receive treatment. Consequent overburdening of HES, combined with under-resourced services may be causing delays in patients being seen and causing harm especially in the higher risk patients (12). Therefore, this bottleneck urgently needs addressing. We propose to mitigate this risk of harm to patients by stratifying patients referred to HES according to their risk of requiring treatment or losing their vision using a clinical prediction model. This would enable higher risk patients to be prioritised and seen sooner.

Clinical prediction models are statistical models that use multiple predictor variables to predict the risk of a clinical outcome (13). They can be used by clinicians to stratify care by risk groups based on the predicted probabilities from the model. The DESP uses risk stratification studies to inform suitable screening intervals (14). There are also prediction models to identify patients at the highest risk of developing referable DR (10, 15, 16), validated in a UK population (17). However, there are currently no such validated prediction models that can be used to stratify care according to risk in patients under the care of HES. Once validated, the DRPTVL-UK model could help HES prioritise patients at high risk of vision loss, and to determine suitable follow up intervals based on an individual's risk.

The current length of follow up intervals used within HES is based on the probability of disease progression from a study conducted in the late eighties (18) and not based on the patient's individual risk. We therefore aim to predict the progression of DR to treatment stage, to direct resources toward higher-risk patients so that they are followed-up and treated before vision failure occurs. We propose that use of a validated risk prediction model will facilitate evidence-based decisions and thus reduce the chance of harm to higher risk patients.

There are two recent systematic reviews of existing models for predicting the progression of DR among the DESP population (15, 19). A review by this group of researchers found a total of 14 predictive model development studies of which 11 had been internally validated, 8 had been externally validated and only three without risk of bias (15). In a more recent review, there were 16 model development studies for an outcome of referable DR (20). Based on these two reviews, it was concluded that none of the existing models were intended for our target population (patients under care of HES / surveillance clinics) and none used the

For peer review only - http://bmjopen.bmj.com/site/about/guidelines.xhtml

clinically important outcomes of interest (including contemporary treatment modalities and vision loss). Therefore, a prediction model that could be used to identify patients with a higher probability of requiring treatment or at high risk of loss of vision for HES was needed.

In brief, the DRPTVL-UK model was developed in anonymised, retrospective primary care data from IQVIA Medical Research Data (IMRD). From predictors identified in our systematic review (15), we selected a set of 19 clinically meaningful candidate predictors of DR progression using the Nominal Group Technique (NGT) (21). A prediction model was then developed considering 15 of the 19 candidate predictors for inclusion based on availability in the dataset (22). After variable selection, the final model included 7 predictors, namely 1) retinopathy stage, 2) HbA1c, 3) eGFR, 4) total serum cholesterol, 5) systolic blood pressure, and drug use of 6) insulin or 7) statins. The DRPTVL-UK model demonstrated moderately good discriminative performance (C-statistic = 0.74) and very little optimism (0.004) in the internal validation due to the large sample size (13,691 patients).

a) Rationale

We now need to assess the model's predictive performance in a secondary care population to ensure it performs adequately to identify patients at high risk of treatment or vision loss in a HES setting. If this model performs well for predicting risk at time points up to two years in the external validation using HES / surveillance clinic data, we propose that it could be used to prioritise individuals at higher risk of vision loss and potentially inform the length of the follow up intervals after referral to HES / surveillance clinics.

b) Objectives and outcome measures/endpoints

The overall aim is to externally validate the multivariable risk prediction model we previously developed, recalibrating to the secondary care population if necessary and updating with additional predictor variables if necessary. The primary objectives are to:

- Assess the external validity of the DRPTVL-UK model for predicting the risk of need for treatment or vision loss up to 2 years after referral in a hospital-based DR population by assessing model calibration, discrimination and net benefit.
- Evaluate whether recalibration of the baseline hazard or linear predictor (combination of predictor effects) improves predictive performance in a HES / surveillance clinic population and whether including additional predictors improves the model's predictive performance in this population.

Secondary objectives are to:

For peer review only - http://bmjopen.bmj.com/site/about/guidelines.xhtml

DRPTVL-UK Model validation

- Assess the DRPTVL-UK model's external validity in the subgroup of patients with pre-proliferative DR (R2) or M1.
- Validate the model across several time points up to 2 years to assess whether it could be used to inform follow-up intervals after referral into HES or a surveillance clinic.

Parallel with the validation, there will be two consensus meetings of expert clinicians and patients to determine how the model can be implemented in practice and establish clinically meaningful thresholds for use.

METHODS AND ANALYSIS

For peer review only

This prognostic study has been guided by the PROGRESS framework (theme 3 for prognostic models) and will be reported according to the TRIPOD Statement (for Transparent Reporting of a multivariable prediction model for individual prognosis or diagnosis) (23).

Design and data sources

This retrospective cohort study will use patient data collected from HES/Surveillance clinic and other related databases/patient notes from three NHS trusts. Where required information is not available for extraction from the hospitals' notes, it will be obtained from surveillance clinics if available (and vice versa) by the participating NHS trusts.

Patients and public involvement

Patients and the public have been involved in the design of this study (see Appendix 1 for details), and will be involved in the conduct, reporting and dissemination of this research.

Study population

The cohort will include type 1 and type 2 diabetic patients, aged 12 years and over (as patients enter the screening programme from age 12), referred into HES or surveillance clinics with referable DR from DESP for close monitoring and treatment. Data will be collected from three NHS trusts for all patients within the catchment areas of Sandwell and Birmingham, Sunderland, or Sussex NHS trusts. Records will be extracted for patients first entering the services between 1st January 2013 and 31st December 2016, with follow up information extracted up to 31st December 2021. The Birmingham trust cares for an ethnically and socio-economically diverse range of communities and was chosen to ensure

For peer review only - http://bmjopen.bmj.com/site/about/guidelines.xhtml

equality, diversity, and inclusion. The Sussex trust provides secondary care to a less diverse population and Sunderland is a primarily Caucasian population.

Patients with the specific outcome of retinopathy treatment or vision loss at referral or those referred for reasons other than retinopathy will be excluded. Patients objecting to their information being used (through a local or national opt out scheme) will also be excluded. Data flow and management stages are given in figure 2 below.

Sample size

A minimum of 200 outcome events are required for external validation using current guidance for survival outcomes (24). Every trust receives approximately 200 referrals per year and we expect to have 4 years' worth of data available for each trust. Therefore, we expect to have approximately 2,400 patients from across the three trusts to ensure a minimum of 200 outcomes. Recruitment will stop once the target number is reached.

Using conservative estimates from our development data, we expect 15% of those referred to develop the outcome of interest within 3 years follow up (22), providing at least 360 outcomes in the data that we will collect. For model updating, we will use the method of Riley et al. (25) to calculate the minimum sample size required, assuming an event rate of 0.05 per year, mean follow up of 3.23 years, a default Nagelkerke R^2 of 0.15 and 19 candidate predictors considered in the model. A minimum of 1810 patients are required with 293 outcome events to target a shrinkage factor of 0.9 ensuring minimal overfitting to the data.

Data extraction

A data extraction sheet has been prepared using Microsoft Excel and will be piloted prior to use within each trust. Data will be extracted for all predictors at baseline (first HES appointment after referral) and visual acuity level will be collected at each follow up appointment. Data will continue to be extracted until an outcome of interest occurs, death, loss to follow up or the end of the study period (31st December 2021).

Personal information such as date of birth (for age calculation), date of diabetes diagnosis and date of death (for censoring or competing risks analysis) will be extracted. However, all the personal identifiable information will be removed after age, diabetes duration and follow-up durations have been calculated.

Outcomes

For peer review only - http://bmjopen.bmj.com/site/about/guidelines.xhtml

DRPTVL-UK Model validation

The primary outcome for this study is time to first treatment for DR or vision loss. Time will be calculated from referral to HES (baseline is first appointment) until the date of first treatment or vision loss. Time to death will also be recorded and treated as a competing risk if necessary.

Predictor variables

The previously developed DRPTVL-UK model included 7 predictors measured at the time of or close to referral, namely 1) retinopathy stage, 2) HbA1c (mmol/mol), 3) eGFR (ml min^{-1} 1.73), 4) total serum cholesterol (mmol/l), 5) systolic blood pressure (mm Hg), and drug use of 6) insulin for 7) statins.

Some candidate predictors like the Early Worsening, high non-attendance rate, Pregnancy and Visual acuity were not previously available in the IMRD database used to develop the model but will be collected from the three trusts. Their definitions are in appendices 2 and 3.

DRPTVL-UK model

We previously developed and internally validated the DRPTVL-UK for the purpose of predicting the risk of vision loss and blindness or need for treatment in patients with referable diabetic retinopathy. The model had good discrimination (C-statistic=0.74) and very little optimism in the internal validation. The model (22) was developed in a primary care population and now needs external validation in the hospital eye service/surveillance clinic population, in which it is intended to be used.

The DRPTVL-UK model was developed using Cox regression and later refitted using a flexible parametric approach to obtain the baseline hazard function over time. The model can be used to predict the absolute risk of progression from referable DR to treatment or vision loss occurring within a 2-year period, based on an individual's risk factor values. Thorough evaluation of the model's external validity and net benefit is now required to establish whether the model is suitable for use in clinical practice in hospital eye services/surveillance clinics.

The DRPTVL-UK model can also be used to predict the time at which an individual reaches a particular risk threshold (to be agreed in a consensus meeting of clinical experts and patients planned after final analysis) which may be useful for determining appropriate follow-up intervals after referral to hospital eye services/surveillance clinics. This will be evaluated

Main Text Document

For peer review only - http://bmjopen.bmj.com/site/about/guidelines.xhtml

as a secondary objective.

External validation of the DRPTVL-UK model

The DRPTVL-UK model will be used to obtain the predicted probability of the outcome over time for every participant within each of the three trusts. Predictive performance of the model will be assessed using measures of discrimination (Harrell's C-statistic and time-dependent C-statistic), calibration (calibration slope, ratio of Observed to Expected probabilities, calibration plots at multiple time points up to 2 years) and net benefit using decision curves. Performance measures will be calculated within each hospital and then pooled on an appropriate scale using random effects meta-analysis to account for clustering by hospital (26) .

As another potential use of the model would be to determine appropriate follow-up intervals based on the individual's risk, it will also be crucial to ensure that the model performs well for predictions at all time points up to two years to ensure risk predictions are accurate over time. Therefore, we will also evaluate calibration performance at multiple time points. In addition to this, we will look at the predictions over time (predicted survival curves) and compare these to the observed survival curves for risk groups and other meaningful groupings e.g., DR grade.

In addition to external validation of the model in the whole sample, we will also validate the model within the subgroups of R2 / M1 patients to see how well it performs in each.

Summarising baseline variables

Baseline variables will include predictors measured at referral or shortly before (see Table 1 for full list of variables to be collected). Continuous variables will be described using means and SDs (prior to centring and standardisation), binary or categorical variables will be described using frequencies and proportions.

Table 1: List of variables for data collection - predictors modified from (21), Outcomes and competing risk variables.

For peer review only - http://bmjopen.bmj.com/site/about/guidelines.xhtml

DRPTVL-UK Model validation

	Group	Required variables	Source, units
1	*Ocular features	Diabetic retinopathy grade	DESP / Hospital Notes / letter
2		Visual acuity score	Both eyes, Log MAR, every visit with date till the outcome
3	Biochemical parameters	HbA1c	From Biochemistry database (mmol/mol)
4		eGFR	From Biochemistry database, ml min^{-1} 1.73
5		Total Serum Cholesterol	From Biochemistry database, mmol/l
6	Physical examination	Systolic Blood pressure	From nursing notes, mm Hg
7	Diabetes treatment	Statin	From GP letter / Diabetology notes
8		Insulin	From GP letter / Diabetology notes
9	*NGT	Pregnancy	During the preceding 2 years before referral
10		Early worsening	To be calculated at baseline (details in appendices 2 and 3).
11		Frequent DNA / cancellations (total, two consecutive sets)	With dates (Patient Administration System)
12	Competing risk	Date of death if occurring before the treatment / vision failure / date of discharge	Patient Administration System, deidentify after
13	Outcome / follow up	Date of treatment (first ever) / vision failure / discharge / transfer / end of the study – End Dec 2021 (whichever happens first)	Patient notes
14	Demographics	Age, gender, ethnicity and deprivation score	Patient notes

*Among the predictors, ocular features (DR stage in each eye) and Visual acuity will be

Main Text Document

For peer review only

recorded for both eyes at every visit along with the date of measurement. For analysis, the eye with the higher DR grade will be used (R3M1>R3>R2M1>M1>R2). For other predictors in the model, values up to and including the first referral (baseline) appointment will be used, provided they occur in the 12 months prior to the referral appointment. The measurement closest to the referral (baseline) appointment will be used. Please also see appendices 2 & 3 for details.

Model recalibration and updating

If necessary, we will recalibrate the model to the HES population (for example, by updating the baseline survival function or recalibrating the linear predictor) (27). Additionally, we will investigate whether updating the model to include additional predictors that were not available in the development dataset improves the predictive performance. Visual acuity, early worsening, pregnancy, and frequent “did not attend” (DNA), were identified as candidate predictors based on expert opinion and evidence evaluation (21). To update the model with additional predictors, flexible parametric models (Royston-Parmar models) will be fitted using a multivariable fractional polynomial (MFP) approach to consider non-linear functions for continuous variables while using backward elimination for the additional predictors considered (28). We will use a p-value > 0.157 as a proxy for selection based on Akaike Information Criterion (29). All predictors from the original model will be forced to remain in the model regardless of statistical significance, therefore only the four additional variables will be tested. The predictive performance of the updated model will be evaluated using internal-external cross-validation (30) in which the model is developed using the data from two hospitals and externally validated in the third. This is then repeated a total of three times, each time reserving a different hospital for external validation. Predictive performance will be evaluated in each ‘external’ hospital using the same measures of discrimination and calibration as previously described and will be summarised across the hospitals using random-effects meta-analysis. Predictive performance of the updated model will be compared to the original model.

Clinical Benefit

We will also evaluate the clinical benefit of the model using decision curve analysis in which the net benefit of using the model at different threshold probabilities is plotted and compared to strategies of intervention for all (following up everyone more frequently) or intervention for no-one (no-one followed up more frequently) (31).

For peer review only - http://bmjopen.bmj.com/site/about/guidelines.xhtml

DRPTVL-UK Model validation

Missing Data

Missing data is a common problem in clinical data and needs to be appropriately accounted for in analyses. An audit using hospital notes from Sunderland Eye Infirmary showed physical examination variables of systolic and diastolic blood pressure nearest to referral were recorded in the clinical notes of 72% of patients; biochemical variables of HbA1c were recorded for 83% of patients, eGFR and cholesterol in 95.5% of patients, measured near to referral. In each case, we will consider why the values might be missing to understand whether a missing at random assumption is reasonable or whether missingness is likely to be informative. For variables missing for <40% of patients, missing data will be handled by multiple imputation using chained equations assuming data are missing at random. The missing at random assumption is an untestable one but data checks comparing characteristics of patients with missing values to those without will be performed to assess if there are any obvious problems with the assumption. To preserve any clustering that may be present, data will be imputed for each hospital separately. The imputation model will include all predictors as well as the outcome using the event indicator and estimate of the cumulative hazard function. Auxiliary variables will be considered to improve the missing at random assumption. The number of imputed datasets will be set at least equal to the percentage of observations of missing data for any of the variables of interest (32).

Clinical consensus

Alongside the statistical analysis, clinical consensus workshops will take place to help determine clinically meaningful threshold probabilities for net benefit analysis and for use of the model in practice. This will include discussion and agreement on a suitable threshold for identifying higher risk patients and potential thresholds for determining the follow-up intervals, prior to decision curve analysis. After the results of the external validation including the decision curve analysis are available, they will be presented to the ophthalmic expert committee panel for discussion on how the model can be implemented.

The consensus process was first used in the United States of America in the early 1970s to address the National Institutes of Health development programme to seek agreement on the safety and efficacy of medical procedures, drugs and devices (33). Consensus development meetings were introduced to the UK health system to discuss healthcare policies and its implementation in clinical practice (34)

The consensus process will be used in this expert group to reduce the range of potential options presented to facilitate joint decision-making by the group on the most appropriate choice of the model implementation strategies. The consensus process will help us evaluate

For peer review only - http://bmjopen.bmj.com/site/about/guidelines.xhtml

the list of options and combine them if an overlap is noted between different options. It can also accommodate the inclusion of further options, check for redundancy between included options and reach agreement through sharing information and knowledge of the participants (35). The consensus process described below also enhances the critical thinking of the key stakeholders and facilitates joint decision-making of the diverse groups (36). Communication and cooperation between participants are key to reach successful agreement on the options discussed and to increase the chances of wider acceptance for implementation (37). Here we aim to reach an agreement on participants' opinions on the various options under consideration.

In this study participants will be asked to rate the importance of each of the options based on a nine-point Likert scale that has been adopted in the COMET consensus style; (1-3 = less important, 4-6 = important and 7-9 = critical) using a 70% threshold agreement to score the quality of evidence for outcomes in systematic reviews, and has been adopted in other core outcome development research groups using Delphi methods (38). Therefore, participants will be asked to vote on whether particular risk groupings should be considered for the final DRPTVL-UK model, excluded, or require further discussion and refinement. For each option presented, the proportion of participants scoring 1-3, 4-6 and 7-9 on the nine-point Likert scale will be calculated for each item. "Consensus in" will be defined as greater than 70% of participants scoring as 7-9. 'Consensus Out' is based on an item being scored 1-3 by more than 70%. No consensus is based on an item where the level of importance was not decided due to uncertainty (39). We anticipate that the group joining the consensus process will include 10-15 participants, ensuring an appropriate balance of representation of the different stake holder groups.

For peer review only

Software

All statistical analysis will be done using Stata 16 and R version 4.1 or later versions.

Ethics

DRPTVL-UK Model validation

For the external validation, routine practice retrospective data including the lists of all NHS numbers of referrals for the years 2013 to 2016 will be kept on NHS trusts' R & D secure computers in lockable rooms. Population characteristics like age at baseline/ date of birth for age range, date of death for competing risk analysis will be extracted, but after calculation of age, gender distribution and lengths of follow up durations, all the personal identifiable information will be removed to protect the patient privacy/confidentiality. Data extractors will be clinical nurse specialists employed by the NHS trusts' R & D. No sensitive data will be extracted. Identifiable data will be removed from data extracted and only anonymised data will be sent to University of Birmingham (UoB) in encrypted fashion. This anonymised data will be stored on secure UoB computers with appropriate access controls, in lockable rooms.

For the consensus process, write ups will not mention any direct quotes, the NHS trust or any individual expert's identity. This anonymised data will be stored on secure NHS / UoB computers for 10 years to allow for all possible publications.

Personal identifiable data will be deleted using deletion software at the time of study end by the contributing NHS trusts. Small number suppression (≤7, as per NHS Digital practice for Hospital Episode Statistics Admitted Patient Care data) will apply (40).

Dissemination

The plans for dissemination include peer reviewed publication, presentation to professional / PPIE bodies and development of an electronic calculator application to allow risk-based prioritization of their follow up, after direct entry of clinical information. However, further research may be required to assess the clinical and economic impact of the final model. Social media will also be used to disseminate findings.

LIMITATIONS

Missing data is the main limitation foreseen. Small sample size for subgroup analysis may reduce discrimination ability.

CONCLUSIONS

There is need for risk-based prioritisation for high-risk patients to reduce their risk of harm. Accurate risk stratification can be an effective mechanism to help decision making in these critical scenarios. However, there is currently no high-quality risk assessment mechanism specific to these high-risk patients under care of hospital eye services. The planned study aims to fulfil the need for an accurate and implementable risk prediction model for these patients.

Main Text Document

For peer review only

DRPTVL-UK Model validation

Authors affiliations

1. Institute of Applied Health Research, University of Birmingham, Birmingham, UK
2. Sandwell and West Birmingham Hospitals NHS Trust, Birmingham, UK
3. Sunderland Eye Infirmary, Queen Alexandra Rd, Sunderland UK
4. Bioscience Institute, Newcastle University, Newcastle Upon Tyne, UK
5. Surrey and Sussex Hospitals NHS Trust, Redhill, UK
6. Centre for Prognosis Research, School of Medicine, Keele University, Staffordshire, UK

Twitter SajjadH78235604

Authors' contributions:

SH and KIES designed the main study. MT led the consensus and co-led PPIE along with SH. SS will lead the data extraction. SH and KIES drafted the protocol manuscript. DHS, IS and RC contributed substantially towards the concept and secondary objectives write up. NJA, KIES, KN, NIHR panel, Ethics committee members, Confidentiality Advisory Group and members of the ophthalmic expert committee provided valuable input during the funding application and project approvals. All of the authors read, gave feedback on the manuscript and approved the final version.

Funding

This project is funded by the National Institute for Health and Care Research (NIHR) under its Research for Patient Benefit (RfPB) Programme (Grant Reference Number NIHR203608). The views expressed are those of the author(s) and not necessarily those of the NIHR or the Department of Health and Social Care.

Competing interests

The authors have no competing interest to disclose.

Patient consent for publication Not required.

Ethics approval

Research ethics committee and CAG approval for use of support under section 251 support to process confidential patient information without consent. There is no direct patient contact as the study is retrospective, therefore consent taking is not going to be possible.

Main Text Document

DRPTVL-UK Model validation

Open Access

This is an open access article distributed in accordance with the Creative Commons Attribution Non Commercial (CC BY-NC 4.0) license, which permits others to distribute, remix, adapt, build upon this work non-commercially, and license their derivative works on different terms, provided the original work is properly cited, appropriate credit is given, any changes made indicated, and the use is non-commercial. See: http://creativecommons.org/licenses/by-nc/4.0/

ORCID iD

Sajjad Haider 0000-0001-8111-8577
Kym I. E. Snell 0000-0001-9373-6591
David H Steel 0000-0001-8734-3089

Research reference numbers

1. Funder's reference number (NIHR RfPB) - NIHR203608
2. Sponsor University of Birmingham, reference number – RG_22-099
3. IRAS reference number – 253774

For peer review only - http://bmjopen.bmj.com/site/about/guidelines.xhtml

DRPTVL-UK Model validation

References

1. Diabetes UK. Diabetes diagnoses double in the last 15 years 2021 [Available from: https://www.diabetes.org.uk/about_us/news/diabetes-diagnoses-doubled-prevalence-2021.
2. Haider S, Thayakaran R, Subramanian A, Toulis KA, Moore D, Price MJ, et al. Disease burden of diabetes, diabetic retinopathy and their future projections in the UK: cross-sectional analyses of a primary care database. BMJ open. 2021;11(7):e050058.
3. (NCD-RisC) NRFC. Worldwide trends in diabetes since 1980: a pooled analysis of 751 population-based studies with 4.4 million participants. Lancet. 2016;387(10027):1513-30.
4. Gonzalez EL, Johansson S, Wallander MA, Rodriguez LA. Trends in the prevalence and incidence of diabetes in the UK: 1996-2005. Journal of epidemiology and community health. 2009;63(4):332-6.
5. Public Health England. Diabetes Prevalence Model 2016 [Available from: https://assets.publishing.service.gov.uk/government/uploads/system/uploads/attachment_data/file/612306/Diabetesprevalencemodelbriefing.pdf, Accessed 2019, July 12.
6. Mathur R, Bhaskaran K, Edwards E, Lee H, Chaturvedi N, Smeeth L, et al. Population trends in the 10-year incidence and prevalence of diabetic retinopathy in the UK: a cohort study in the Clinical Practice Research Datalink 2004-2014. BMJ open. 2017;7(2):e014444.
7. UK D. NHS spending on diabetes 'to reach £16.9 billion by 2035 2021 [Available from: https://www.diabetes.org.uk/about_us/news_landing_page/nhs-spending-on-diabetes-to-reach-169-billion-by-2035.
8. Fowler MJ. Microvascular and Macrovascular Complications of Diabetes. Clinical Diabetes. 2008;26(2):77-82.
9. Bourne RRA, Jonas JB, Bron AM, Cicinelli MV, Das A, Flaxman SR, et al. Prevalence and causes of vision loss in high-income countries and in Eastern and Central Europe in 2015: magnitude, temporal trends and projections. The British journal of ophthalmology. 2018;102(5):575-85.
10. Scanlon PH, Aldington SJ, Leal J, Luengo-Fernandez R, Oke J, Sivaprasad S, et al. Development of a cost-effectiveness model for optimisation of the screening interval in diabetic retinopathy screening. Health Technol Assess. 2015;19(74):1-116.
11. Jyothi S, Elahi B, Srivastava A, Poole M, Nagi D, Sivaprasad S. Compliance with the quality standards of National Diabetic Retinopathy Screening Committee. Primary care diabetes. 2009;3(2):67-72.
12. The Royal College of Ophthalmologists. Surveillance of Sight Loss due to delay in ophthalmic review in the UK: Frequency, cause and outcome 2015 [Available from: https://www.rcophth.ac.uk/standards-publications-research/audit-and-data/the-british-ophthalmological-surveillance-unit-bosu/abstract-surveillance-of-sight-loss-due-to-delay-in-ophthalmic-review-in-the-uk/.
13. Steyerberg EW, Moons KG, van der Windt DA, Hayden JA, Perel P, Schroter S, et al. Prognosis Research Strategy (PROGRESS) 3: prognostic model research. PLoS medicine. 2013;10(2):e1001381.

Main Text Document

For peer review only

14. Scanlon PH. Screening Intervals for Diabetic Retinopathy and Implications for Care. Current Diabetes Reports. 2017;17(10):96.
15. Haider S, Sadiq SN, Moore D, Price MJ, Nirantharakumar K. Prognostic prediction models for diabetic retinopathy progression: a systematic review. Eye. 2019;33(5):702-13.
16. Aspelund T, Porisdottir O, Olafsdottir E, Gudmundsdottir A, Einarsdottir AB, Mehlsen J, et al. Individual risk assessment and information technology to optimise screening frequency for diabetic retinopathy. Diabetologia. 2011;54(10):2525-32.
17. Lund SH, Aspelund T, Kirby P, Russell G, Einarsson S, Palsson O, et al. Individualised risk assessment for diabetic retinopathy and optimisation of screening intervals: A scientific approach to reducing healthcare costs. British Journal of Ophthalmology. 2016;100(5):683-7.
18. EDTRS. Fundus photographic risk factors for progression of diabetic retinopathy. ETDRS report number 12. Early Treatment Diabetic Retinopathy Study Research Group. Ophthalmology. 1991;98(5 Suppl):823-33.
19. van der Heijden A, Badloe F, Nijpels G, Beulens J. Prediction models for the risk of retinopathy in people with type 2 diabetes. A systematic review. European Journal of Ophthalmology. 2017;27(3):E117-E.
20. van der Heijden AA, Nijpels G, Badloe F, Lovejoy HL, Peelen LM, Feenstra TL, et al. Prediction models for development of retinopathy in people with type 2 diabetes: systematic review and external validation in a Dutch primary care setting. Diabetologia. 2020;63(6):1110-9.
21. Haider S, Sadiq SN, Lufumpa E, Sihre H, Tallouzi M, Moore DJ, et al. Predictors for diabetic retinopathy progression—findings from nominal group technique and Evidence review. BMJ Open Ophthalmology. 2020;5(1):e000579.
22. Haider S. Optimisation of Health Services for Sight Threatening Diabetic Retinopathy in the UK: University of Birmingham 2020.
23. Collins GS, Reitsma JB, Altman DG, Moons KG. Transparent Reporting of a multivariable prediction model for Individual Prognosis Or Diagnosis (TRIPOD): the TRIPOD Statement. The British journal of surgery. 2015;102(3):148-58.
24. Vergouwe Y, Steyerberg EW, Eijkemans MJ, Habbema JD. Substantial effective sample sizes were required for external validation studies of predictive logistic regression models. Journal of clinical epidemiology. 2005;58(5):475-83.
25. Riley RD, Snell KI, Ensor J, Burke DL, Harrell FE, Jr., Moons KG, et al. Minimum sample size for developing a multivariable prediction model: PART II - binary and time-to-event outcomes. Stat Med. 2019;38(7):1276-96.
26. Snell KI, Ensor J, Debray TP, Moons KG, Riley RD. Meta-analysis of prediction model performance across multiple studies: Which scale helps ensure between-study normality for the C-statistic and calibration measures? Statistical Methods in Medical Research. 2018;27(11):3505-22.
27. Ensor J, Snell KIE, Debray TPA, Lambert PC, Look MP, Mamas MA, et al. Individual participant data meta-analysis for external validation, recalibration, and updating of a flexible parametric prognostic model. Statistics in Medicine. 2021;40(13):3066-84.

For peer review only - http://bmjopen.bmj.com/site/about/guidelines.xhtml

28. Riley RD, van der Windt D, Croft P, Moons KGM. Prognosis Research in Health Care: Concepts, Methods, and Impact: Oxford University Press; 2019.
29. Heinze G, Wallisch C, Dunkler D. Variable selection - A review and recommendations for the practicing statistician. Biom J. 2018;60(3):431-49.
30. Debray TP, Moons KG, Ahmed I, Koffijberg H, Riley RD. A framework for developing, implementing, and evaluating clinical prediction models in an individual participant data meta-analysis. Stat Med. 2013;32(18):3158-80.
31. Vickers AJ, Elkin EB. Decision curve analysis: a novel method for evaluating prediction models. Med Decis Making. 2006;26(6):565-74.
32. White IR, Royston P, Wood AM. Multiple imputation using chained equations: Issues and guidance for practice. Stat Med. 2011;30(4):377-99.
33. Goodman C BS, editors,. Improving Consensus Development for Health Technology Assessment: An International Perspective. The National Institutes of Health Office of Medical Applications of Research1990.
34. R O C K W E L L S C H U L Z and S T E V E H A R R I S O N. Consensus Management in the British National Health Service: Implications for the United States? [Available from: https://www.milbank.org/wp-content/uploads/mq/volume-62/issue-04/62-4-Consensus-Management-in-the-British-National-Health-Service.pdf.
35. Duffy J, Rolph R, Gale C, Hirsch M, Khan KS, Ziebland S, et al. Core outcome sets in women's and newborn health: a systematic review. Bjog. 2017;124(10):1481-9.
36. Schlessinger DI, Iyengar S, Yanes AF, Lazaroff JM, Godinez-Puig V, Chen BR, et al. Development of a core outcome set for clinical trials in basal cell carcinoma: study protocol for a systematic review of the literature and identification of a core outcome set using a Delphi survey. Trials. 2017;18(1):490.
37. Steurer J. The Delphi method: an efficient procedure to generate knowledge. Skeletal Radiology. 2011;40(8):959-61.
38. GRADE Working Group. Grading of recommendations assessment, development and evaluation (GRADE). 2012.
39. Harman NL, Bruce IA, Callery P, Tierney S, Sharif MO, O'Brien K, et al. MOMENT–Management of Otitis Media with Effusion in Cleft Palate: protocol for a systematic review of the literature and identification of a core outcome set using a Delphi survey. Trials. 2013;14(1):1.
40. Digital. N. NHS Digital. Data Quality Maturity Index (DQMI) methodology. Suppression rules. 2021, 2021 [cited 2022 november]. Available from: https://digital.nhs.uk/data-and-information/data-tools-and-services/data-services/data-quality/data-quality-maturity-index-methodology/suppression-rules.

For peer review only - http://bmjopen.bmj.com/site/about/guidelines.xhtml

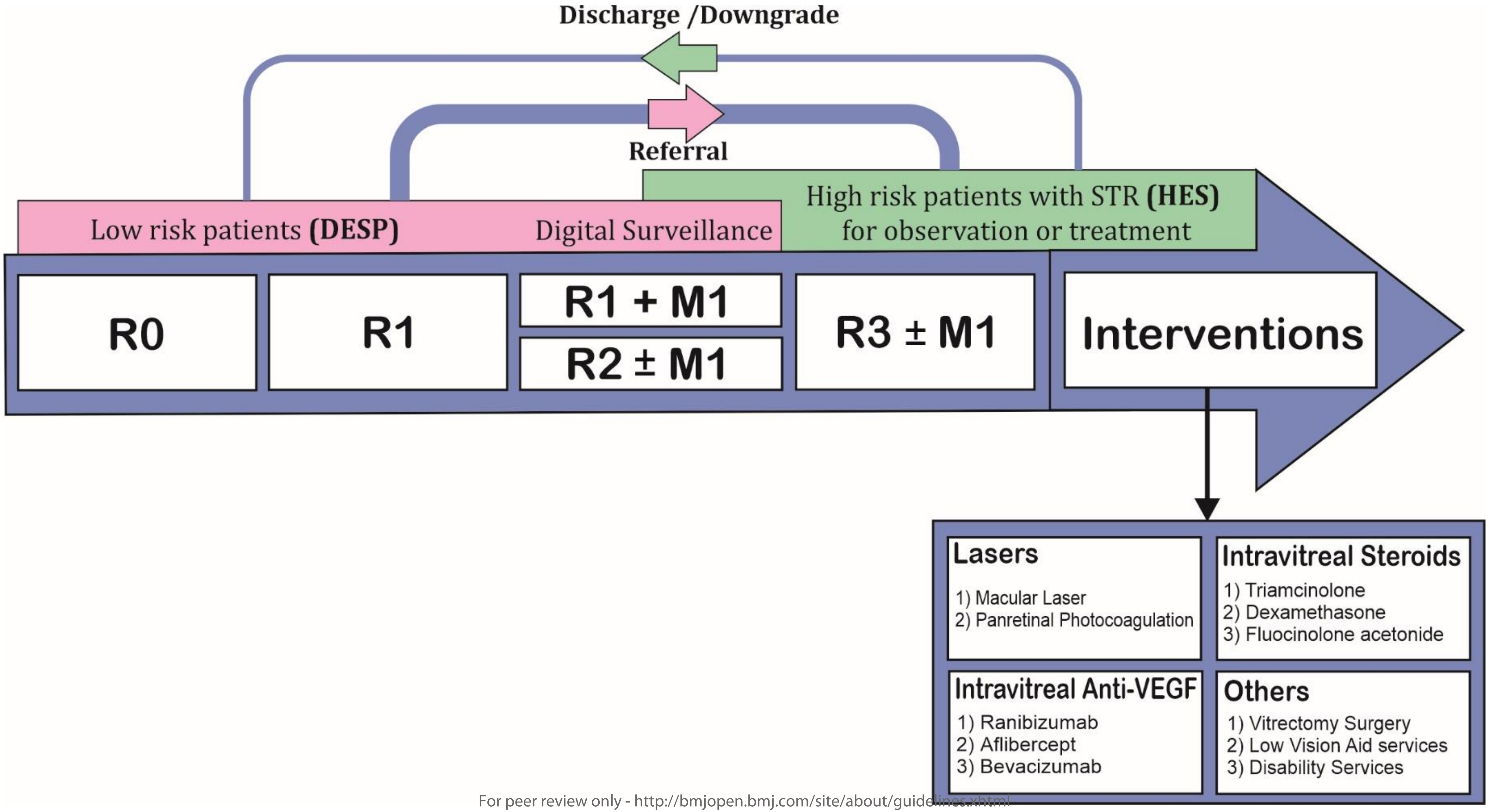
Discharge /Downgrade
Referral
Low risk patients (DESP)
Digital Surveillance
High risk patients with STR (HES)
for observation or treatment
R0
R1
R1 + M1
R2 ± M1
R3 ± M1
Interventions
Lasers
1) Macular Laser
2) Panretinal Photocoagulation
Intravitreal Steroids
1) Triamcinolone
2) Dexamethasone
3) Fluocinolone acetonide
Intravitreal Anti-VEGF
1) Ranibizumab
2) Aflibercept
3) Bevacizumab
Others
1) Vitrectomy Surgery
2) Low Vision Aid services
3) Disability Services

A. Identification of study participants

- Data extractor team in the three NHS trusts will work with the Diabetic Eye Screening Programme (DESP) and hospital eye service to obtain the list of all the referrals from DESP to surveillance clinics / hospital eye service between Jan 2013 to Dec 2016.

B. Finalisation of study participants

- Electronic and/or paper clinic notes will be reviewed by the clinical nurse specialist with help of local data coordinators / local PI
- Exclusion criteria will be applied and list of study participants will be finalised

C. Data Extraction

- Data extraction excel sheet will be generated. Clinical nurse specialist from each trust will extract data on the list of variables using electronic and /or paper clinical notes. STS NHS trust will require CAG support for patient identifiable data extraction.
- Master copy will be stored on local NHS Trust R & D secure computers

D. Anonymisation

- Anonymised copy of data extraction sheet will be generated by the local data coordinator with the help of information governance using the master copy.
- All patient identifiable information will be removed and data completely anonymised

E. Data Extraction Monitoring

Anonymsed data will be sent to the designated data extraction coordinator at trust level using secure nhs.net email (Shahina Ahmed in SASH, Mohammad Tallouzi in SWBH and Salman Sadiq in STS will support clinical nurse specialists in their respective NHS trusts). The trust data extraction coordinators will review data on regular basis and feedback to data extraction nurse. Further training will be provided by the central data extraction coordinator(Salman Sadiq) as necessary.

F. Data Transfer

Anonymised data will then be sent to the University of Birmingham for analysis on monthly basis in an encrypted fashion using secure nhs.net email Only members of the research team will have access to the data received?

G. Data Storage and Disposal

- University stores the data on a secure local network and will ensure secure storage for ten years.
- Local R&D teams will dispose of master copy of data from each trust soomn after analysis finishes.

For peer review only - http://bmjopen.bmj.com/site/about/guidelines.xhtml

Appendices

Appendix 1: Pre-application patients and public involvement and engagement (PPIE) and later consultation with stake holders

The author has previously published on the predictors for progression of DR according to the James Lind Alliance priority setting (1) (priority 3 under retinal vascular disease/sight loss and vision) (2). The present study addresses priority 8 on the same top 10 research priorities (barriers that prevent diabetic patients having regular eye checks). For wider clinical expert input, we held consultation meetings with ophthalmologist colleagues with DR as their special interest, their DESP colleagues, diabetologists interested in DR and GP's with specialist interest in diabetes. They all provided detailed written feedback which was incorporated into the research design for funding application. The table below summarises the PPIE activities undertaken.

Table : List of PPIE activities (mostly pre-application)

For peer review only

DRPTVL-UK Model validation

PPIE Activity Report - Modified from GRIPP 2 (3)		
1	**Aims**	The aim of this PPIE exercise was to get patients' perspective about this research and to involve them in the design of the study and in the grant application.
2	**Objectives**	To recruit a diverse group for equitable representation.
		To train patients with diabetic retinopathy under care of the hospital eye services joining the group in PPIE.
		To ensure the use of friendly and plain language in the lay summary.
		To get PPIE input into the research project.
		To form a patient steering group and to recruit a patient advocate as a co- applicant with a deputy.
3	**Methods**	Recruitment through Diabetes UK, Clinical Research Network, three NHS trusts (northeast, midlands and southeast), local research networks, and through GP forums in order to include a diverse group and to ensure equitable access. The patients had been living with diabetic retinopathy and had been under the care of hospital eye services for at least one year.
		A presentation on all aspects of the research followed by questions and answers followed by open ended discussion
		Requested a volunteer to help write the plain English summary.
		In the presentation, we explained important themes of the research design and plans, but also ensured an adequate open-ended discussion to cover unforeseen patient perspectives, experiences, and concerns. We then brainstormed to gain further patient input.
		We invited two volunteers to act as co-applicants as patient advocates.
4	**Study results**	The patient advisory meeting was held remotely on 4th March 2021. 8 participants (including a GP representative) from three different regions of various ages and of different ethnicities attended. Patients without any internet access were invited into a GP practice to provide access to the virtual meeting.

Supplementary File

DRPTVL-UK Model validation

		Participants reviewed the presentation, asked questions, engaged in discussion, and responded to the meeting minutes. They were supportive of the research and felt it will be beneficial for patients.
		One patient revised the summary to make it easier to read through user-friendly language.
		There were two important comments from patients on study design. They wanted to ensure safety for the patients where model does not accurately predict and did not want the ceiling for follow-up intervals to be as high as 2 years as in Diabetic Eye Screening Programme.
		Two volunteers accepted the invitation to act as patient advocates, one as co-applicant and the other as deputy. The group also agreed to be part of patient steering group and play a key role in disseminating the results of the study to the public, patients, their families, and carers.
5	**Discussion and conclusions**	Patients' perspectives regarding the follow up intervals, to be designed up to a maximum of 2 years, was taken on board. The risk arising from uncertainty in the model predictions will be mitigated by raising this issue in the consensus meeting for further discussion before finalising outputs.
6	**Reflections/critical perspective**	A PPIE group comprising a relevant population is now in place. This needs to grow in size for sustainability.

PPIE activity report (Modified from GRIPP 2, Short form https://doi.org/10.1136/bmj.j3453)

After receiving the feedback from NIHR panel on the stage 1 application, we discussed various comments with an ophthalmic expert panel. We have incorporated their advice into this protocol, added a secondary objectives section and added further analysis to external validation in the methods section.

Supplementary File

For peer review only - http://bmjopen.bmj.com/site/about/guidelines.xhtml

DRPTVL-UK Model validation

Before starting the data permission applications and regulatory approvals, we tested the acceptability of using anonymised patient data in this study without consent by sending an e mail to the group asking this question. Following were the responses received.

1) "I can't foresee any issues with using patient data so long as it has been completely anonymised".
2) "I am happy for mine to be used".
3) "I think it's a great idea and helps with the project".

Appendix 2: Feasibility of NGT suggested variables:

Early worsening of DR (EWDR)

EWDR arises within 6 months after abrupt improvement of glucose control (> 4% or > 20.2 mmol/mol, during intensive treatment - insulin pump therapy and after pancreas transplantation or bariatric surgery). Follow up is required over the following 12 months. EWDR is often transient, with regression of retinal signs after 12 months in the Oslo study in all except four patients [8] and in nearly half of the DCCT patients (4). We shall extract HBA1c twice before baseline.

Audit data from a trust contributing data, impression was that this variable is not well recorded. From prediction point of view, we can look at feasibility of it once data is available. If feasible, then can include it in the model to see if it makes a difference to the model performance. For the patients with the outcomes of treatment, we shall look back at the last 12/12 of HBA1c levels and the evidence of intensive treatment, bariatric / pancreatic surgery.

Pregnancy: While pregnancy is associated with progression of diabetic retinopathy (5), and in type 1, it induces a transient increase (2.5-fold) in the risk of retinopathy (6). There is also a low risk of progression of DR in type 2 diabetes (7) as well. Increased ophthalmic surveillance is needed during pregnancy and the first year postpartum. From modelling perspective this variable may not be relevant as most patients are beyond reproductive age (> 60 years mean). But we shall use the variable as history of pregnancy less than two years before the outcome of need for treatment.

Supplementary File

For peer review only - http://bmjopen.bmj.com/site/about/guidelines.xhtml

Frequent DNA / Cancellations

Patients with history of non-attendance of diabetic eye screening for two consecutive years are at increased risk of developing Sight Threatening Diabetic Retinopathy (8). Evidence of this in patients with referral retinopathy under care of surveillance clinic or hospital services does not exist. However this was voted 8th out of 33 predictors in a nominal group technique exercise (2) attended by ophthalmic clinicians. We shall use this variable during the external update of the model. We shall collect data on total number of non-attendance and cancellations and no of > 1 consecutive non-attendance or cancellations before the baseline (first HES appointment records).

Appendix 3: Important definitions

- Early worsening: "DR progression to treatment requiring stage during the first year after rapid improvement in blood glucose will be considered EWDR" if there is history of intensive treatment / bariatric / pancreatic surgery (4).
- Follow up: From the first appointment after referral by DESP to first treatment (laser / injection) or vision failure, whichever comes first, death, discharge, transfer or end of the study.
- Outcome: This is a composite of treatment (photocoagulation, injection, vitrectomy) or vision failure (vision loss or blindness)
- Treatment: photocoagulation, Intraocular injection treatment with any anti VEGF or steroid injections laser or vitreous surgery
- Vision failure: Loss of three lines of vision (10 to 15 letters on EDTRS) or more, only if it happens before treatment. (Conversions-Between-Letter-LogMAR-and-Snellen-Visual-Acuity-Scores.png (605×725) (researchgate.net), (VALIDITY OF OUTCOME MEASURES - Ranibizumab (Lucentis) - NCBI Bookshelf (nih.gov)

For peer review only - http://bmjopen.bmj.com/site/about/guidelines.xhtml

DRPTVL-UK Model validation

References

1. James Lind Alliance. Priority Setting Partnerships, Retinal Vascular Disease Top 10 [cited 2013. Available from: https://www.jla.nihr.ac.uk/priority-setting-partnerships/sight-loss-and-vision/top-10-priorities/retinal-vascular-disease-top-10.htm.
2. Haider S, Sadiq SN, Lufumpa E, Sihre H, Tallouzi M, Moore DJ, et al. Predictors for diabetic retinopathy progression—findings from nominal group technique and Evidence review. BMJ Open Ophthalmology. 2020;5(1):e000579.
3. Staniszewska S, Brett J, Simera I, Seers K, Mockford C, Goodlad S, et al. GRIPP2 reporting checklists: tools to improve reporting of patient and public involvement in research. BMJ. 2017;358:j3453.
4. Feldman-Billard S, Larger E, Massin P. Early worsening of diabetic retinopathy after rapid improvement of blood glucose control in patients with diabetes. Diabetes Metab. 2018;44(1):4-14.
5. Klein BE, Moss SE, Klein R. Effect of pregnancy on progression of diabetic retinopathy. Diabetes care. 1990;13(1):34-40.
6. DCCT. Effect of pregnancy on microvascular complications in the diabetes control and complications trial. The Diabetes Control and Complications Trial Research Group. Diabetes care. 2000;23(8):1084-91.
7. Rasmussen KL, Laugesen CS, Ringholm L, Vestgaard M, Damm P, Mathiesen ER. Progression of diabetic retinopathy during pregnancy in women with type 2 diabetes. Diabetologia. 2010;53(6):1076-83.
8. Forster AS, Forbes A, Dodhia H, Connor C, Du Chemin A, Sivaprasad S, et al. Non-attendance at diabetic eye screening and risk of sight-threatening diabetic retinopathy: a population-based cohort study. Diabetologia. 2013;56(10):2187-93.

Supplementary File

For peer review only - http://bmjopen.bmj.com/site/about/guidelines.xhtml

BMJ Open

Diabetic Retinopathy Progression in patients under monitoring for treatment or vision loss: External Validation, and update of a Multivariable Prediction Model.

Journal:	*BMJ Open*
Manuscript ID	bmjopen-2023-073015.R1
Article Type:	Protocol
Date Submitted by the Author:	16-Mar-2023

Complete List of Authors:	Haider, Sajjad; University of Birmingham, Institute of Applied Health Research<br>Adderley, Nicola; University of Birmingham, Institute of Applied Health Research<br>Tallouzi, Mohammad O.; Birmingham and Midland Eye Centre<br>Sadiq, Salman ; Royal Victoria Infirmary, Ophthalmology; Sunderland Eye Infirmary,<br>Steel, David; Sunderland Eye Infirmary; Newcastle University Biosciences Institute<br>Chavan, Randhir; Sandwell and West Birmingham Hospitals NHS Trust<br>Sheikh, Ijaz; Surrey and Sussex Healthcare NHS Trust<br>Nirantharakumar, Krishnarajah; University of Birmingham, Institute of Applied Health Research<br>Snell, Kym IE; Keele University, Centre for Prognosis Research, School of Medicine, Keele University

**Primary Subject Heading**:	Ophthalmology
Secondary Subject Heading:	Diabetes and endocrinology, Ophthalmology
Keywords:	Diabetic retinopathy < DIABETES & ENDOCRINOLOGY, Medical retina < OPHTHALMOLOGY, Ophthalmology < SURGERY, DIABETES & ENDOCRINOLOGY

SCHOLARONE™
Manuscripts

DRPTVL-UK Model validation

Diabetic Retinopathy Progression in patients under monitoring for treatment or vision loss: External Validation, and update of a Multivariable Prediction Model.

Sajjad Haider[1], Nicola J. Adderley[1], Mohammad O. Tallouzi[1,2] Salman Naveed Sadiq[3], David H. Steel[3,4], Randhir Chavan[2], Ijaz Sheikh[5], Krishnarajah Nirantharakumar[1,] Kym I. E. Snell[6]

Corresponding author:

Sajjad Haider, Institute of Applied Health Research, University of Birmingham, Birmingham, United Kingdom

E mail: S.Haider.2@bham.ac.uk

Authors affiliations

1. Institute of Applied Health Research, University of Birmingham, Birmingham, UK
2. Sandwell and West Birmingham Hospitals NHS Trust, Birmingham, UK
3. Sunderland Eye Infirmary, Queen Alexandra Rd, Sunderland UK
4. Bioscience Institute, Newcastle University, Newcastle Upon Tyne, UK
5. Surrey and Sussex Hospitals NHS Trust, Redhill, UK
6. Centre for Prognosis Research, School of Medicine, Keele University, Staffordshire, UK

ABSTRACT

Introduction

The number of people with diabetes mellitus is increasing globally and consequently so too is diabetic retinopathy (DR). Most diabetic patients are monitored through the diabetic eye screening programme (DESP) until they have signs of retinopathy and these changes progress, requiring referral into hospital eye services (HES). Here they continue to be monitored until they require treatment. Due to current pressures on HES, delays can occur, leading to harm. There is a need to triage patients based on their individual risk. At present, patients are stratified according to retinopathy stage alone, yet other risk factors like HBA1c may be useful. Therefore, a prediction model that combines multiple prognostic factors to predict progression will be useful for triage in this setting to improve care.

We previously developed a Diabetic Retinopathy Progression model to Treatment or Vision Loss (DRPTVL-UK) using a large primary care database. The aim of the present study is to externally validate the DRPTVL-UK model in a secondary care setting, specifically in a population under care by HES. This study will also provide an opportunity to update the model by considering additional predictors not previously available.

Amended Main Text Document

For peer review only

Methods and analysis

We will use a retrospective cohort of 2400 diabetic patients aged 12 years and over, referred from DESP to the NHS hospital trusts with referable DR between 2013 and 2016, with follow-up information recorded until December 2021.
We will evaluate the external validity of the DRPTVL-UK model using measures of discrimination, calibration and net benefit. Additionally, consensus meetings will be held to agree on acceptable risk thresholds for triage within the HES system.

Ethics and Dissemination

This study was approved by REC (ref 22/SC/0425, 05/12/2022, Hampshire A Research Ethics Committee).
Registration: ISRCTN 10956293
The results of the study will be published in a peer-reviewed journal, presented at clinical conferences.

For peer review only

Strengths and limitations of this study

- The model will be externally validated in the secondary care setting in which it is intended to be used.
- Extracting the data (not using an existing dataset) ensures the sample size will be large enough for the aims.
- Retrospective cohort design means data can be extracted relatively quickly at minimal cost.
- Opportunity to update the model to include additional predictors which may improve predictive performance.
- As data are being collected retrospectively, some predictor variables and outcomes may be missing for some patients or trusts. This will be dealt with by using multiple imputation where possible.

INTRODUCTION

Diabetes mellitus is one of the most common chronic conditions affecting nearly 4.8 million people in UK as of 2019 (1). With the prevalence rising each year (2), there is an ongoing global and UK wide increase in the number of people with diabetes mellitus (3-5) and

For peer review only - http://bmjopen.bmj.com/site/about/guidelines.xhtml

consequently diabetic retinopathy DR. Our study estimated about 1.4 million with any DR and 0.54 million with referrable stage DR (2) in 2017. The detection of DR has also improved through wider population screening, further increasing the demand for HES (6). Diabetes is a major public health concern and uses a significant proportion of the NHS budget, much of which is spent treating the complications of diabetes (7). These complications affect blood vessels in the heart, brain, kidney and eyes (8). Diabetes is the fourth leading cause of preventable vision loss in the UK (9), and therefore patients with diabetes are screened regularly for signs of DR. Screening services are organised by the Diabetic Eye Screening Programme (DESP) for patients without DR or with background DR. However, when a patient develops clinical signs of referable retinopathy, including pre-proliferative diabetic retinopathy (R2), proliferative diabetic retinopathy (R3) and / or diabetic maculopathy (M1), they are referred to HES or surveillance clinics for closer observation and treatment to prevent vision loss. The patients' flow within the NHS is depicted in the figure 1.

Most referrals made to HES (50 to 78%) will not yet require treatment (10, 11). Among those that will require treatment, such as patients with diabetic maculopathy, the patients' condition may be subthreshold (under 400 microns foveal thickness) for treatment and remain so for a period of time. Patients with pre-proliferative retinopathy are not offered any treatment and are monitored every three to six months until they progress to the proliferative retinopathy stage, at which point they receive treatment. Consequent overburdening of HES, combined with under-resourced services may be causing delays in patients being seen and causing harm especially in the higher risk patients (12). Therefore, this bottleneck urgently needs addressing. We propose to mitigate this risk of harm to patients by stratifying patients referred to HES according to their risk of requiring treatment or losing their vision using a clinical prediction model. This would enable higher risk patients to be prioritised and seen sooner.

Clinical prediction models are statistical models that use multiple predictor variables to predict the risk of a clinical outcome (13). They can be used by clinicians to stratify care by risk groups based on the predicted probabilities from the model. The DESP uses risk stratification studies to inform suitable screening intervals (14). There are also prediction models to identify patients at the highest risk of developing referable DR (10, 15, 16), validated in a UK population (17). However, there are currently no such validated prediction models that can be used to stratify care according to risk in patients under the care of HES. Once validated, the DRPTVL-UK model could help HES prioritise patients at high risk of vision loss, and to determine suitable follow up intervals based on an individual's risk.

For peer review only - http://bmjopen.bmj.com/site/about/guidelines.xhtml

DRPTVL-UK Model validation

The current length of follow up intervals used within HES is based on the probability of disease progression from a study conducted in the late eighties (18) and not based on the patient's individual risk. We therefore aim to predict the progression of DR to treatment stage, to direct resources toward higher-risk patients so that they are followed-up and treated before vision failure occurs. We propose that use of a validated risk prediction model will facilitate evidence-based decisions and thus reduce the chance of harm to higher risk patients.

There are two recent systematic reviews of existing models for predicting the progression of DR among the DESP population (15, 19). A review by this group of researchers found a total of 14 predictive model development studies of which 11 had been internally validated, 8 had been externally validated and only three without risk of bias (15). In a more recent review, there were 16 model development studies for an outcome of referable DR (20). Based on these two reviews, it was concluded that none of the existing models were intended for our target population (patients under care of HES / surveillance clinics) and none used the clinically important outcomes of interest (including contemporary treatment modalities and vision loss). Therefore, a prediction model that could be used to identify patients with a higher probability of requiring treatment or at high risk of loss of vision for HES was needed.

In brief, the DRPTVL-UK model was developed in anonymised, retrospective primary care data from IQVIA Medical Research Data (IMRD). From predictors identified in our systematic review (15), we selected a set of 19 clinically meaningful candidate predictors of DR progression using the Nominal Group Technique (NGT) (21). A prediction model was then developed considering 15 of the 19 candidate predictors for inclusion based on availability in the dataset (22). After variable selection, the final model included 7 predictors, namely 1) retinopathy stage, 2) HbA1c, 3) eGFR, 4) total serum cholesterol, 5) systolic blood pressure, and drug use of 6) insulin or 7) statins. The DRPTVL-UK model demonstrated moderately good discriminative performance (C-statistic = 0.74) and very little optimism (0.004) in the internal validation due to the large sample size (13,691 patients).

a) Rationale

We now need to assess the model's predictive performance in a secondary care population to ensure it performs adequately to identify patients at high risk of treatment or vision loss in a HES setting. If this model performs well for predicting risk at time points up to two years in the external validation using HES / surveillance clinic data, we propose that it could be used to prioritise individuals at higher risk of vision loss and potentially inform the length of the follow up intervals after referral to HES / surveillance clinics.

Amended Main Text Document

For peer review only

b) Objectives and outcome measures/endpoints

The overall aim is to externally validate the multivariable risk prediction model we previously developed, recalibrating to the secondary care population if necessary and updating with additional predictor variables if necessary. The primary objectives are to:

- Assess the external validity of the DRPTVL-UK model for predicting the risk of need for treatment or vision loss up to 2 years after referral in a hospital-based DR population by assessing model calibration, discrimination and net benefit.
- Evaluate whether recalibration of the baseline hazard or linear predictor (combination of predictor effects) improves predictive performance in a HES / surveillance clinic population and whether including additional predictors improves the model's predictive performance in this population.

Secondary objectives are to:

- Assess the DRPTVL-UK model's external validity in the subgroup of patients with pre-proliferative DR (R2) or M1.
- Validate the model across several time points up to 2 years to assess whether it could be used to inform follow-up intervals after referral into HES or a surveillance clinic.

Parallel with the validation, there will be two consensus meetings of expert clinicians and patients to determine how the model can be implemented in practice and establish clinically meaningful thresholds for use.

METHODS AND ANALYSIS

This prognostic study has been guided by the PROGRESS framework (theme 3 for prognostic models) and will be reported according to the TRIPOD Statement (for Transparent Reporting of a multivariable prediction model for individual prognosis or diagnosis) (23). The study started 1st July 2022, with data extraction planned to start 1st of April 2023. Study is scheduled to end by the end of December 2023.

Design and data sources

This retrospective cohort study will use patient data collected from HES/Surveillance clinic and other related databases/patient notes from three NHS trusts. Where required information is not available for extraction from the hospitals' notes, it will be obtained from

For peer review only - http://bmjopen.bmj.com/site/about/guidelines.xhtml

surveillance clinics if available (and vice versa) by the participating NHS trusts.

Patients and public involvement

Patients and the public have been involved in the design of this study (see Appendix 1 for details), and will be involved in the conduct, reporting and dissemination of this research.

Study population

The cohort will include type 1 and type 2 diabetic patients, aged 12 years and over (as patients enter the screening programme from age 12), referred into HES or surveillance clinics with referable DR from DESP for close monitoring and treatment. Data will be collected from three NHS trusts for all patients within the catchment areas of Sandwell and Birmingham, Sunderland, or Sussex NHS trusts. Records will be extracted for patients first entering the services between 1st January 2013 and 31st December 2016, with follow up information extracted up to 31st December 2021. The Birmingham trust cares for an ethnically and socio-economically diverse range of communities and was chosen to ensure equality, diversity, and inclusion. The Sussex trust provides secondary care to a less diverse population and Sunderland is a primarily Caucasian population.

Patients with the specific outcome of retinopathy treatment or vision loss at referral or those referred for reasons other than retinopathy will be excluded. Patients objecting to their information being used (through a local or national opt out scheme) will also be excluded. Data flow and management stages are given in figure 2 below.

Sample size

A minimum of 200 outcome events are required for external validation using current guidance for survival outcomes (24). Every trust receives approximately 200 referrals per year and we expect to have 4 years' worth of data available for each trust. Therefore, we expect to have approximately 2,400 patients from across the three trusts to ensure a minimum of 200 outcomes. Recruitment will stop once the target number is reached.

Using conservative estimates from our development data, we expect 15% of those referred to develop the outcome of interest within 3 years follow up (22), providing at least 360 outcomes in the data that we will collect. For model updating, we will use the method of Riley et al. (25) to calculate the minimum sample size required, assuming an event rate of 0.05 per year, mean follow up of 3.23 years, a default Nagelkerke R^2 of 0.15 and 19 candidate predictors considered in the model. A minimum of 1810 patients are required with 293 outcome events to target a shrinkage factor of 0.9 ensuring minimal overfitting to the data.

For peer review only - http://bmjopen.bmj.com/site/about/guidelines.xhtml

DRPTVL-UK Model validation

Data extraction

A data extraction sheet has been prepared using Microsoft Excel and will be piloted prior to use within each trust. Data will be extracted for all predictors at baseline (first HES appointment after referral) and visual acuity level will be collected at each follow up appointment. Data will continue to be extracted until an outcome of interest occurs, death, loss to follow up or the end of the study period (31st December 2021).

Personal information such as date of birth (for age calculation), date of diabetes diagnosis and date of death (for censoring or competing risks analysis) will be extracted. However, all the personal identifiable information will be removed after age, diabetes duration and follow-up durations have been calculated.

Outcomes

The primary outcome for this study is time to first treatment for DR or vision loss. Time will be calculated from referral to HES (baseline is first appointment) until the date of first treatment or vision loss. Time to death will also be recorded and treated as a competing risk if necessary.

Predictor variables

The previously developed DRPTVL-UK model included 7 predictors measured at the time of or close to referral, namely 1) retinopathy stage, 2) HbA1c (mmol/mol), 3) eGFR (ml min^{-1} 1.73), 4) total serum cholesterol (mmol/l), 5) systolic blood pressure (mm Hg), and drug use of 6) insulin for 7) statins.

Some candidate predictors like the Early Worsening, high non-attendance rate, Pregnancy and Visual acuity were not previously available in the IMRD database used to develop the model but will be collected from the three trusts. Their definitions are in appendices 2 and 3.

DRPTVL-UK model

We previously developed and internally validated the DRPTVL-UK for the purpose of predicting the risk of vision loss and blindness or need for treatment in patients with referable diabetic retinopathy. The model had good discrimination (C-statistic=0.74) and very little optimism in the internal validation. The model (22) was developed in a primary care population and now needs external validation in the hospital eye service/surveillance clinic

Amended Main Text Document

For peer review only - http://bmjopen.bmj.com/site/about/guidelines.xhtml

population, in which it is intended to be used.

The DRPTVL-UK model was developed using Cox regression and later refitted using a flexible parametric approach to obtain the baseline hazard function over time. The model can be used to predict the absolute risk of progression from referable DR to treatment or vision loss occurring within a 2-year period, based on an individual's risk factor values. Thorough evaluation of the model's external validity and net benefit is now required to establish whether the model is suitable for use in clinical practice in hospital eye services/surveillance clinics.

The DRPTVL-UK model can also be used to predict the time at which an individual reaches a particular risk threshold (to be agreed in a consensus meeting of clinical experts and patients planned after final analysis) which may be useful for determining appropriate follow-up intervals after referral to hospital eye services/surveillance clinics. This will be evaluated as a secondary objective.

External validation of the DRPTVL-UK model

The DRPTVL-UK model will be used to obtain the predicted probability of the outcome over time for every participant within each of the three trusts. Predictive performance of the model will be assessed using measures of discrimination (Harrell's C-statistic and time-dependent C-statistic), calibration (calibration slope, ratio of Observed to Expected probabilities, calibration plots at multiple time points up to 2 years) and net benefit using decision curves. Performance measures will be calculated within each hospital and then pooled on an appropriate scale using random effects meta-analysis to account for clustering by hospital (26) .

As another potential use of the model would be to determine appropriate follow-up intervals based on the individual's risk, it will also be crucial to ensure that the model performs well for predictions at all time points up to two years to ensure risk predictions are accurate over time. Therefore, we will also evaluate calibration performance at multiple time points. In addition to this, we will look at the predictions over time (predicted survival curves) and compare these to the observed survival curves for risk groups and other meaningful groupings e.g., DR grade.

In addition to external validation of the model in the whole sample, we will also validate the model within the subgroups of R2 / M1 patients to see how well it performs in each.

For peer review only - http://bmjopen.bmj.com/site/about/guidelines.xhtml

DRPTVL-UK Model validation

Summarising baseline variables

	Group	Required variables	Source, units
1	*Ocular features	Diabetic retinopathy grade	DESP / Hospital Notes / letter
2		Visual acuity score	Both eyes, Log MAR, every visit with date till the outcome
3	Biochemical parameters	HbA1c	From Biochemistry database (mmol/mol)
4		eGFR	From Biochemistry database, ml min^{-1} 1.73
5		Total Serum Cholesterol	From Biochemistry database, mmol/l
6	Physical examination	Systolic Blood pressure	From nursing notes, mm Hg
7	Diabetes treatment	Statin	From GP letter / Diabetology notes
8		Insulin	From GP letter / Diabetology notes
9	*NGT	Pregnancy	During the preceding 2 years before referral

Baseline variables will include predictors measured at referral or shortly before (see Table 1 for full list of variables to be collected). Continuous variables will be described using means and SDs (prior to centring and standardisation), binary or categorical variables will be described using frequencies and proportions.

Table 1: List of variables for data collection - predictors modified from (21), Outcomes and competing risk variables.

For peer review only - http://bmjopen.bmj.com/site/about/guidelines.xhtml

10		Early worsening	To be calculated at baseline (details in appendices 2 and 3).
11		Frequent DNA / cancellations (total, two consecutive sets)	With dates (Patient Administration System)
12	Competing risk	Date of death if occurring before the treatment / vision failure / date of discharge	Patient Administration System, deidentify after
13	Outcome / follow up	Date of treatment (first ever) / vision failure / discharge / transfer / end of the study – End Dec 2021 (whichever happens first)	Patient notes
14	Demographics	Age, gender, ethnicity and deprivation score	Patient notes

*Among the predictors, ocular features (DR stage in each eye) and Visual acuity will be recorded for both eyes at every visit along with the date of measurement. For analysis, the eye with the higher DR grade will be used (R3M1>R3>R2M1>M1>R2). For other predictors in the model, values up to and including the first referral (baseline) appointment will be used, provided they occur in the 12 months prior to the referral appointment. The measurement closest to the referral (baseline) appointment will be used. Please also see appendices 2 & 3 for details.

Model recalibration and updating

If necessary, we will recalibrate the model to the HES population (for example, by updating the baseline survival function or recalibrating the linear predictor) (27). Additionally, we will investigate whether updating the model to include additional predictors that were not available in the development dataset improves the predictive performance. Visual acuity, early worsening, pregnancy, and frequent "did not attend" (DNA), were identified as candidate predictors based on expert opinion and evidence evaluation (21). To update the model with additional predictors, flexible parametric models (Royston-Parmar models) will be fitted using a multivariable fractional polynomial (MFP) approach to consider non-linear functions for continuous variables while using backward elimination for the additional predictors considered (28). We will use a p-value > 0.157 as a proxy for selection based on Akaike Information Criterion (29). All predictors from the original model will be forced to remain in the model regardless of statistical significance, therefore only the four additional

For peer review only - http://bmjopen.bmj.com/site/about/guidelines.xhtml

variables will be tested. The predictive performance of the updated model will be evaluated using internal-external cross-validation (30) in which the model is developed using the data from two hospitals and externally validated in the third. This is then repeated a total of three times, each time reserving a different hospital for external validation. Predictive performance will be evaluated in each 'external' hospital using the same measures of discrimination and calibration as previously described and will be summarised across the hospitals using random-effects meta-analysis. Predictive performance of the updated model will be compared to the original model.

Clinical Benefit

We will also evaluate the clinical benefit of the model using decision curve analysis in which the net benefit of using the model at different threshold probabilities is plotted and compared to strategies of intervention for all (following up everyone more frequently) or intervention for no-one (no-one followed up more frequently) (31).

Missing Data

Missing data is a common problem in clinical data and needs to be appropriately accounted for in analyses. An audit using hospital notes from Sunderland Eye Infirmary showed physical examination variables of systolic and diastolic blood pressure nearest to referral were recorded in the clinical notes of 72% of patients; biochemical variables of HbA1c were recorded for 83% of patients, eGFR and cholesterol in 95.5% of patients, measured near to referral. In each case, we will consider why the values might be missing to understand whether a missing at random assumption is reasonable or whether missingness is likely to be informative. For variables missing for <40% of patients, missing data will be handled by multiple imputation using chained equations assuming data are missing at random. The missing at random assumption is an untestable one but data checks comparing characteristics of patients with missing values to those without will be performed to assess if there are any obvious problems with the assumption. To preserve any clustering that may be present, data will be imputed for each hospital separately. The imputation model will include all predictors as well as the outcome using the event indicator and estimate of the cumulative hazard function. Auxiliary variables will be considered to improve the missing at random assumption. The number of imputed datasets will be set at least equal to the percentage of observations of missing data for any of the variables of interest (32).

Clinical consensus

Alongside the statistical analysis, clinical consensus workshops will take place to help

For peer review only - http://bmjopen.bmj.com/site/about/guidelines.xhtml

determine clinically meaningful threshold probabilities for net benefit analysis and for use of the model in practice. This will include discussion and agreement on a suitable threshold for identifying higher risk patients and potential thresholds for determining the follow-up intervals, prior to decision curve analysis. After the results of the external validation including the decision curve analysis are available, they will be presented to the ophthalmic expert committee panel for discussion on how the model can be implemented.

The consensus process was first used in the United States of America in the early 1970s to address the National Institutes of Health development programme to seek agreement on the safety and efficacy of medical procedures, drugs and devices (33). Consensus development meetings were introduced to the UK health system to discuss healthcare policies and its implementation in clinical practice (34)

The consensus process will be used in this expert group to reduce the range of potential options presented to facilitate joint decision-making by the group on the most appropriate choice of the model implementation strategies. The consensus process will help us evaluate the list of options and combine them if an overlap is noted between different options. It can also accommodate the inclusion of further options, check for redundancy between included options and reach agreement through sharing information and knowledge of the participants (35). The consensus process described below also enhances the critical thinking of the key stakeholders and facilitates joint decision-making of the diverse groups (36). Communication and cooperation between participants are key to reach successful agreement on the options discussed and to increase the chances of wider acceptance for implementation (37). Here we aim to reach an agreement on participants' opinions on the various options under consideration.

In this study participants will be asked to rate the importance of each of the options based on a nine-point Likert scale that has been adopted in the COMET consensus style; (1-3 = less important, 4-6 = important and 7-9 = critical) using a 70% threshold agreement to score the quality of evidence for outcomes in systematic reviews, and has been adopted in other core outcome development research groups using Delphi methods (38). Therefore, participants will be asked to vote on whether particular risk groupings should be considered for the final DRPTVL-UK model, excluded, or require further discussion and refinement. For each option presented, the proportion of participants scoring 1-3, 4-6 and 7-9 on the nine-point Likert scale will be calculated for each item. "Consensus in" will be defined as greater than 70% of participants scoring as 7-9. 'Consensus Out' is based on an item being scored 1-3 by more than 70%. No consensus is based on an item where the level of importance was not decided due to uncertainty (39). We anticipate that the group joining the consensus process will

For peer review only - http://bmjopen.bmj.com/site/about/guidelines.xhtml

include 10-15 participants, ensuring an appropriate balance of representation of the different stake holder groups.

Software

All statistical analysis will be done using Stata 16 and R version 4.1 or later versions.

Ethics and dissemination

For the external validation, routine practice retrospective data including the lists of all NHS numbers of referrals for the years 2013 to 2016 will be kept on NHS trusts' R & D secure computers in lockable rooms. Population characteristics like age at baseline/ date of birth for age range, date of death for competing risk analysis will be extracted, but after calculation of age, gender distribution and lengths of follow up durations, all the personal identifiable information will be removed to protect the patient privacy/confidentiality. Data extractors will be clinical nurse specialists employed by the NHS trusts' R & D. No sensitive data will be extracted. Identifiable data will be removed from data extracted and only anonymised data will be sent to University of Birmingham (UoB) in encrypted fashion. This anonymised data will be stored on secure UoB computers with appropriate access controls, in lockable rooms.

Personal identifiable data will be deleted using deletion software at the time of study end by the contributing NHS trusts. Small number suppression (≤7, as per NHS Digital practice for Hospital Episode Statistics Admitted Patient Care data) will apply (40).

For the consensus process, write ups will not mention any direct quotes, the NHS trust or any individual expert's identity. This anonymised data will be stored on secure NHS / UoB computers for 10 years to allow for all possible publications.

The plans for dissemination include peer reviewed publication, presentation to professional / PPIE bodies and development of an electronic calculator application to allow risk-based prioritization of their follow up, after direct entry of clinical information. However, further research may be required to assess the clinical and economic impact of the final model. Social media will also be used to disseminate findings.

LIMITATIONS

Missing data is the main limitation foreseen. Small sample size for subgroup analysis may reduce the model's discrimination ability.

For peer review only - http://bmjopen.bmj.com/site/about/guidelines.xhtml

DRPTVL-UK Model validation

Twitter SajjadH78235604

Authors' contributions:

SH and KIES designed the main study. MT led the consensus and co-led PPIE along with SH. SS will lead the data extraction. SH and KIES drafted the protocol manuscript. DHS, IS and RC contributed substantially towards the concept and secondary objectives write up. NJA, KIES, KN, NIHR panel, Ethics committee members, Confidentiality Advisory Group and members of the ophthalmic expert committee provided valuable input during the funding application and project approvals. All of the authors read, gave feedback on the manuscript and approved the final version.

Funding

This project is funded by the National Institute for Health and Care Research (NIHR) under its Research for Patient Benefit (RfPB) Programme (Grant Reference Number NIHR203608). The views expressed are those of the author(s) and not necessarily those of the NIHR or the Department of Health and Social Care.

Competing interests

The authors have no competing interest to disclose.

Patient consent for publication Not required.

Ethics approval

REC approval (ref 22/SC/0425, 05/12/2022, South Central -Hampshire A Research Ethics Committee) and CAG approval (ref 22/CAG/0149, 09/12/2022) for use of support under section 251, to process confidential patient information without consent were granted.

Open Access

This is an open access article distributed in accordance with the Creative Commons Attribution Non Commercial (CC BY-NC 4.0) license, which permits others to distribute, remix, adapt, build upon this work non-commercially, and license their derivative works on different terms, provided the original work is properly cited, appropriate credit is given, any changes made indicated, and the use is non-commercial. See: http://creativecommons.org/licenses/by-nc/4.0/

Amended Main Text Document

DRPTVL-UK Model validation

ORCID iD

Sajjad Haider 0000-0001-8111-8577

Kym I. E. Snell 0000-0001-9373-6591

David H Steel 0000-0001-8734-3089

Research reference numbers

1. Funder's reference number (NIHR RfPB) - NIHR203608
2. Sponsor University of Birmingham, reference number – RG_22-099
3. IRAS reference number – 253774

Acknowledgments

We would like to acknowledge the contributions of the expert ophthalmic committee that provided valuable advice and feedback leading to this project being funded. Thanks go to Shahina Ahmed (Surrey and Sussex Hospitals NHS Trust, Redhill, UK), Komala Vedivelue (Durham and Darlington Foundation Trust, UK), Ticiana Criddle (Liverpool University Hospitals Foundation Trust, UK), Maged Habib (South Tyneside & Sunderland NHS Foundation Trust, Honorary Clinical Senior Lecturer. Biosciences Institute. Newcastle University, UK), Daniella vaideanu-Collins (James Cook University Hospital, Marton Road, Middlesbrough, UK), Ramu Muniraju (Surrey Diabetic Eye screening programme, UK), Bob Ryder (Sandwell and West Birmingham Hospitals NHS Trust, Birmingham, UK), Ben Field (Faculty of Health & Medical Sciences, University of Surrey, and Department of Diabetes & Endocrinology, Surrey & Sussex Healthcare NHS Trust UK), Ramesh Sivaraj (University Hospital Birmingham Foundation Trust, UK), Khasheen Alam (Burbury Medical centre Birmingham, UK), Manir Aslam (Broadway Health Centre Birmingham UK), Christopher Sainsbury (Applied Health Research University of Birmingham, UK).

We also like to express our gratitude to the expert patients' panel for their involvement and valuable inputs during pre-funding application process (during research design) and during ethics / Confidentiality Advisory Group (CAG) application for approval.

Data availability statement: Data sharing not applicable as no datasets generated and/or analysed for this study.

For peer review only - http://bmjopen.bmj.com/site/about/guidelines.xhtml

DRPTVL-UK Model validation

References

1. Diabetes UK. Diabetes diagnoses double in the last 15 years 2021 [Available from: https://www.diabetes.org.uk/about_us/news/diabetes-diagnoses-doubled-prevalence-2021.
2. Haider S, Thayakaran R, Subramanian A, Toulis KA, Moore D, Price MJ, et al. Disease burden of diabetes, diabetic retinopathy and their future projections in the UK: cross-sectional analyses of a primary care database. BMJ open. 2021;11(7):e050058.
3. (NCD-RisC) NRFC. Worldwide trends in diabetes since 1980: a pooled analysis of 751 population-based studies with 4.4 million participants. Lancet. 2016;387(10027):1513-30.
4. Gonzalez EL, Johansson S, Wallander MA, Rodriguez LA. Trends in the prevalence and incidence of diabetes in the UK: 1996-2005. Journal of epidemiology and community health. 2009;63(4):332-6.
5. Public Health England. Diabetes Prevalence Model 2016 [Available from: https://assets.publishing.service.gov.uk/government/uploads/system/uploads/attachment_data/file/612306/Diabetesprevalencemodelbriefing.pdf, Accessed 2019, July 12.
6. Mathur R, Bhaskaran K, Edwards E, Lee H, Chaturvedi N, Smeeth L, et al. Population trends in the 10-year incidence and prevalence of diabetic retinopathy in the UK: a cohort study in the Clinical Practice Research Datalink 2004-2014. BMJ open. 2017;7(2):e014444.
7. UK D. NHS spending on diabetes 'to reach £16.9 billion by 2035 2021 [Available from: https://www.diabetes.org.uk/about_us/news_landing_page/nhs-spending-on-diabetes-to-reach-169-billion-by-2035.
8. Fowler MJ. Microvascular and Macrovascular Complications of Diabetes. Clinical Diabetes. 2008;26(2):77-82.
9. Bourne RRA, Jonas JB, Bron AM, Cicinelli MV, Das A, Flaxman SR, et al. Prevalence and causes of vision loss in high-income countries and in Eastern and Central Europe in 2015: magnitude, temporal trends and projections. The British journal of ophthalmology. 2018;102(5):575-85.
10. Scanlon PH, Aldington SJ, Leal J, Luengo-Fernandez R, Oke J, Sivaprasad S, et al. Development of a cost-effectiveness model for optimisation of the screening interval in diabetic retinopathy screening. Health Technol Assess. 2015;19(74):1-116.
11. Jyothi S, Elahi B, Srivastava A, Poole M, Nagi D, Sivaprasad S. Compliance with the quality standards of National Diabetic Retinopathy Screening Committee. Primary care diabetes. 2009;3(2):67-72.
12. The Royal College of Ophthalmologists. Surveillance of Sight Loss due to delay in ophthalmic review in the UK: Frequency, cause and outcome 2015 [Available from: https://www.rcophth.ac.uk/standards-publications-research/audit-and-data/the-british-ophthalmological-surveillance-unit-bosu/abstract-surveillance-of-sight-loss-due-to-delay-in-ophthalmic-review-in-the-uk/.
13. Steyerberg EW, Moons KG, van der Windt DA, Hayden JA, Perel P, Schroter S, et al. Prognosis Research Strategy (PROGRESS) 3: prognostic model research. PLoS medicine. 2013;10(2):e1001381.

For peer review only - http://bmjopen.bmj.com/site/about/guidelines.xhtml

14. Scanlon PH. Screening Intervals for Diabetic Retinopathy and Implications for Care. Current Diabetes Reports. 2017;17(10):96.
15. Haider S, Sadiq SN, Moore D, Price MJ, Nirantharakumar K. Prognostic prediction models for diabetic retinopathy progression: a systematic review. Eye. 2019;33(5):702-13.
16. Aspelund T, Porisdottir O, Olafsdottir E, Gudmundsdottir A, Einarsdottir AB, Mehlsen J, et al. Individual risk assessment and information technology to optimise screening frequency for diabetic retinopathy. Diabetologia. 2011;54(10):2525-32.
17. Lund SH, Aspelund T, Kirby P, Russell G, Einarsson S, Palsson O, et al. Individualised risk assessment for diabetic retinopathy and optimisation of screening intervals: A scientific approach to reducing healthcare costs. British Journal of Ophthalmology. 2016;100(5):683-7.
18. EDTRS. Fundus photographic risk factors for progression of diabetic retinopathy. ETDRS report number 12. Early Treatment Diabetic Retinopathy Study Research Group. Ophthalmology. 1991;98(5 Suppl):823-33.
19. van der Heijden A, Badloe F, Nijpels G, Beulens J. Prediction models for the risk of retinopathy in people with type 2 diabetes. A systematic review. European Journal of Ophthalmology. 2017;27(3):E117-E.
20. van der Heijden AA, Nijpels G, Badloe F, Lovejoy HL, Peelen LM, Feenstra TL, et al. Prediction models for development of retinopathy in people with type 2 diabetes: systematic review and external validation in a Dutch primary care setting. Diabetologia. 2020;63(6):1110-9.
21. Haider S, Sadiq SN, Lufumpa E, Sihre H, Tallouzi M, Moore DJ, et al. Predictors for diabetic retinopathy progression—findings from nominal group technique and Evidence review. BMJ Open Ophthalmology. 2020;5(1):e000579.
22. Haider S. Optimisation of Health Services for Sight Threatening Diabetic Retinopathy in the UK: University of Birmingham 2020.
23. Collins GS, Reitsma JB, Altman DG, Moons KG. Transparent Reporting of a multivariable prediction model for Individual Prognosis Or Diagnosis (TRIPOD): the TRIPOD Statement. The British journal of surgery. 2015;102(3):148-58.
24. Vergouwe Y, Steyerberg EW, Eijkemans MJ, Habbema JD. Substantial effective sample sizes were required for external validation studies of predictive logistic regression models. Journal of clinical epidemiology. 2005;58(5):475-83.
25. Riley RD, Snell KI, Ensor J, Burke DL, Harrell FE, Jr., Moons KG, et al. Minimum sample size for developing a multivariable prediction model: PART II - binary and time-to-event outcomes. Stat Med. 2019;38(7):1276-96.
26. Snell KI, Ensor J, Debray TP, Moons KG, Riley RD. Meta-analysis of prediction model performance across multiple studies: Which scale helps ensure between-study normality for the C-statistic and calibration measures? Statistical Methods in Medical Research. 2018;27(11):3505-22.
27. Ensor J, Snell KIE, Debray TPA, Lambert PC, Look MP, Mamas MA, et al. Individual participant data meta-analysis for external validation, recalibration, and updating of a flexible parametric prognostic model. Statistics in Medicine. 2021;40(13):3066-84.

For peer review only - http://bmjopen.bmj.com/site/about/guidelines.xhtml

28. Riley RD, van der Windt D, Croft P, Moons KGM. Prognosis Research in Health Care: Concepts, Methods, and Impact: Oxford University Press; 2019.
29. Heinze G, Wallisch C, Dunkler D. Variable selection - A review and recommendations for the practicing statistician. Biom J. 2018;60(3):431-49.
30. Debray TP, Moons KG, Ahmed I, Koffijberg H, Riley RD. A framework for developing, implementing, and evaluating clinical prediction models in an individual participant data meta-analysis. Stat Med. 2013;32(18):3158-80.
31. Vickers AJ, Elkin EB. Decision curve analysis: a novel method for evaluating prediction models. Med Decis Making. 2006;26(6):565-74.
32. White IR, Royston P, Wood AM. Multiple imputation using chained equations: Issues and guidance for practice. Stat Med. 2011;30(4):377-99.
33. Goodman C BS, editors,. Improving Consensus Development for Health Technology Assessment: An International Perspective. The National Institutes of Health Office of Medical Applications of Research1990.
34. R O C K W E L L S C H U L Z and S T E V E H A R R I S O N. Consensus Management in the British National Health Service: Implications for the United States? [Available from: https://www.milbank.org/wp-content/uploads/mq/volume-62/issue-04/62-4-Consensus-Management-in-the-British-National-Health-Service.pdf.
35. Duffy J, Rolph R, Gale C, Hirsch M, Khan KS, Ziebland S, et al. Core outcome sets in women's and newborn health: a systematic review. Bjog. 2017;124(10):1481-9.
36. Schlessinger DI, Iyengar S, Yanes AF, Lazaroff JM, Godinez-Puig V, Chen BR, et al. Development of a core outcome set for clinical trials in basal cell carcinoma: study protocol for a systematic review of the literature and identification of a core outcome set using a Delphi survey. Trials. 2017;18(1):490.
37. Steurer J. The Delphi method: an efficient procedure to generate knowledge. Skeletal Radiology. 2011;40(8):959-61.
38. GRADE Working Group. Grading of recommendations assessment, development and evaluation (GRADE). 2012.
39. Harman NL, Bruce IA, Callery P, Tierney S, Sharif MO, O’Brien K, et al. MOMENT–Management of Otitis Media with Effusion in Cleft Palate: protocol for a systematic review of the literature and identification of a core outcome set using a Delphi survey. Trials. 2013;14(1):1.
40. Digital. N. NHS Digital. Data Quality Maturity Index (DQMI) methodology. Suppression rules. 2021, 2021 [cited 2022 november]. Available from: https://digital.nhs.uk/data-and-information/data-tools-and-services/data-services/data-quality/data-quality-maturity-index-methodology/suppression-rules.

For peer review only - http://bmjopen.bmj.com/site/about/guidelines.xhtml

DRPTVL-UK Model validation

Figure 1: Patient flow Diagram (There may be local variations in practices)
R0, No retinopathy; R1, Background retinopathy; R2, Pre-proliferative retinopathy; R3, Proliferative retinopathy; M0, No Maculopathy; M1, Maculopathy present; DESP, Diabetic Eye screening programme; HES, Hospital eye services; STR, Sight threatening retinopathy; VEGF (vascular endothelial growth factor).

Figure 2: Data flow Diagram

For peer review only

Amended Main Text Document

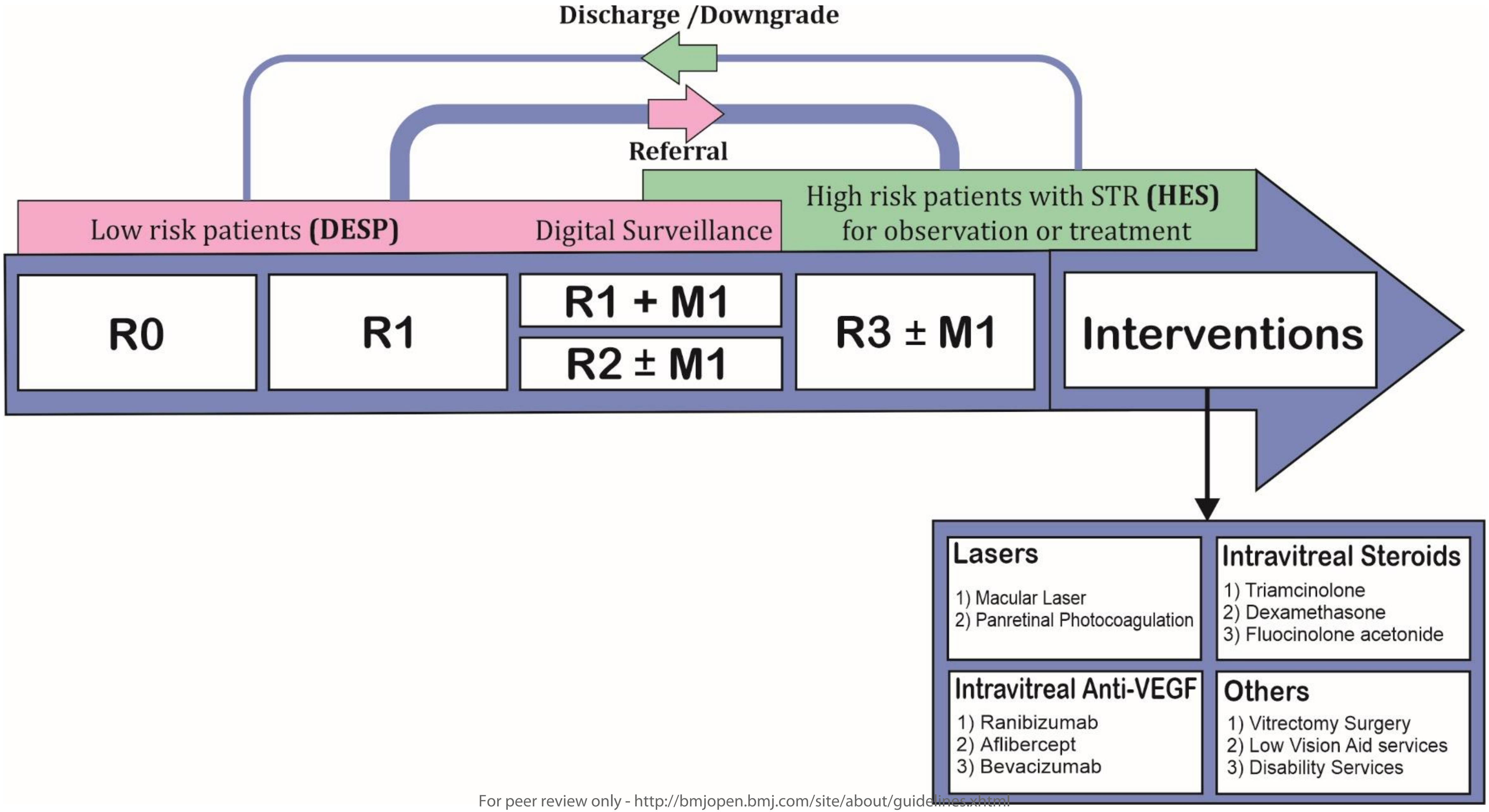

For peer review only - http://bmjopen.bmj.com/site/about/guidelines.xhtml

A. Identification of study participants

- Data extractor team in the three NHS trusts will work with the Diabetic Eye Screening Programme (DESP) and hospital eye service to obtain the list of all the referrals from DESP to surveillance clinics / hospital eye service between Jan 2013 to Dec 2016.

B. Finalisation of study participants

- Electronic and/or paper clinic notes will be reviewed by the clinical nurse specialist with help of local data coordinators / local PI
- Exclusion criteria will be applied and list of study participants will be finalised

C. Data Extraction

- Data extraction excel sheet will be generated. Clinical nurse specialist from each trust will extract data on the list of variables using electronic and /or paper clinical notes. STS NHS trust will require CAG support for patient identifiable data extraction.
- Master copy will be stored on local NHS Trust R & D secure computers

D. Anonymisation

- Anonymised copy of data extraction sheet will be generated by the local data coordinator with the help of information governance using the master copy.
- All patient identifiable information will be removed and data completely anonymised

E. Data Extraction Monitoring

Anonymsed data will be sent to the designated data extraction coordinator at trust level using secure nhs.net email (Shahina Ahmed in SASH, Mohammad Tallouzi in SWBH and Salman Sadiq in STS will support clinical nurse specialists in their respective NHS trusts). The trust data extraction coordinators will review data on regular basis and feedback to data extraction nurse. Further training will be provided by the central data extraction coordinator(Salman Sadiq) as necessary.

F. Data Transfer

Anonymised data will then be sent to the University of Birmingham for analysis on monthly basis in an encrypted fashion using secure nhs.net email Only members of the research team will have access to the data received?

G. Data Storage and Disposal

- University stores the data on a secure local network and will ensure secure storage for ten years.
- Local R&D teams will dispose of master copy of data from each trust soomn after analysis finishes.

DRPTVL-UK Model validation

Appendices

Appendix 1: Pre-application patients and public involvement and engagement (PPIE) and later consultation with stake holders

The author has previously published on the predictors for progression of DR according to the James Lind Alliance priority setting (1) (priority 3 under retinal vascular disease/sight loss and vision) (2). The present study addresses priority 8 on the same top 10 research priorities (barriers that prevent diabetic patients having regular eye checks). For wider clinical expert input, we held consultation meetings with ophthalmologist colleagues with DR as their special interest, their DESP colleagues, diabetologists interested in DR and GP's with specialist interest in diabetes. They all provided detailed written feedback which was incorporated into the research design for funding application. The table below summarises the PPIE activities undertaken.

Table : List of PPIE activities (mostly pre-application)

For peer review only

For peer review only - http://bmjopen.bmj.com/site/about/guidelines.xhtml

DRPTVL-UK Model validation

PPIE Activity Report - Modified from GRIPP 2 (3)		
1	**Aims**	The aim of this PPIE exercise was to get patients' perspective about this research and to involve them in the design of the study and in the grant application.
2	**Objectives**	To recruit a diverse group for equitable representation.
		To train patients with diabetic retinopathy under care of the hospital eye services joining the group in PPIE.
		To ensure the use of friendly and plain language in the lay summary.
		To get PPIE input into the research project.
		To form a patient steering group and to recruit a patient advocate as a co- applicant with a deputy.
3	**Methods**	Recruitment through Diabetes UK, Clinical Research Network, three NHS trusts (northeast, midlands and southeast), local research networks, and through GP forums in order to include a diverse group and to ensure equitable access. The patients had been living with diabetic retinopathy and had been under the care of hospital eye services for at least one year.
		A presentation on all aspects of the research followed by questions and answers followed by open ended discussion
		Requested a volunteer to help write the plain English summary.
		In the presentation, we explained important themes of the research design and plans, but also ensured an adequate open-ended discussion to cover unforeseen patient perspectives, experiences, and concerns. We then brainstormed to gain further patient input.
		We invited two volunteers to act as co-applicants as patient advocates.
4	**Study results**	The patient advisory meeting was held remotely on 4th March 2021. 8 participants (including a GP representative) from three different regions of various ages and of different ethnicities attended. Patients without any internet access were invited into a GP practice to provide access to the virtual meeting.

Supplementary File

DRPTVL-UK Model validation

		Participants reviewed the presentation, asked questions, engaged in discussion, and responded to the meeting minutes. They were supportive of the research and felt it will be beneficial for patients.
		One patient revised the summary to make it easier to read through user-friendly language.
		There were two important comments from patients on study design. They wanted to ensure safety for the patients where model does not accurately predict and did not want the ceiling for follow-up intervals to be as high as 2 years as in Diabetic Eye Screening Programme.
		Two volunteers accepted the invitation to act as patient advocates, one as co-applicant and the other as deputy. The group also agreed to be part of patient steering group and play a key role in disseminating the results of the study to the public, patients, their families, and carers.
5	**Discussion and conclusions**	Patients' perspectives regarding the follow up intervals, to be designed up to a maximum of 2 years, was taken on board. The risk arising from uncertainty in the model predictions will be mitigated by raising this issue in the consensus meeting for further discussion before finalising outputs.
6	**Reflections/critical perspective**	A PPIE group comprising a relevant population is now in place. This needs to grow in size for sustainability.

PPIE activity report (Modified from GRIPP 2, Short form https://doi.org/10.1136/bmj.j3453)

After receiving the feedback from NIHR panel on the stage 1 application, we discussed various comments with an ophthalmic expert panel. We have incorporated their advice into this protocol, added a secondary objectives section and added further analysis to external validation in the methods section.

Supplementary File

DRPTVL-UK Model validation

Before starting the data permission applications and regulatory approvals, we tested the acceptability of using anonymised patient data in this study without consent by sending an e mail to the group asking this question. Following were the responses received.

1) "I can't foresee any issues with using patient data so long as it has been completely anonymised".
2) "I am happy for mine to be used".
3) "I think it's a great idea and helps with the project".

Appendix 2: Feasibility of NGT suggested variables:

Early worsening of DR (EWDR)

EWDR arises within 6 months after abrupt improvement of glucose control (> 4% or > 20.2 mmol/mol, during intensive treatment - insulin pump therapy and after pancreas transplantation or bariatric surgery). Follow up is required over the following 12 months. EWDR is often transient, with regression of retinal signs after 12 months in the Oslo study in all except four patients [8] and in nearly half of the DCCT patients (4). We shall extract HBA1c twice before baseline.

Audit data from a trust contributing data, impression was that this variable is not well recorded. From prediction point of view, we can look at feasibility of it once data is available. If feasible, then can include it in the model to see if it makes a difference to the model performance. For the patients with the outcomes of treatment, we shall look back at the last 12/12 of HBA1c levels and the evidence of intensive treatment, bariatric / pancreatic surgery.

Pregnancy: While pregnancy is associated with progression of diabetic retinopathy (5), and in type 1, it induces a transient increase (2.5-fold) in the risk of retinopathy (6). There is also a low risk of progression of DR in type 2 diabetes (7) as well. Increased ophthalmic surveillance is needed during pregnancy and the first year postpartum. From modelling perspective this variable may not be relevant as most patients are beyond reproductive age (> 60 years mean). But we shall use the variable as history of pregnancy less than two years before the outcome of need for treatment.

Supplementary File

For peer review only

DRPTVL-UK Model validation

Frequent DNA / Cancellations

Patients with history of non-attendance of diabetic eye screening for two consecutive years are at increased risk of developing Sight Threatening Diabetic Retinopathy (8). Evidence of this in patients with referral retinopathy under care of surveillance clinic or hospital services does not exist. However this was voted 8th out of 33 predictors in a nominal group technique exercise (2) attended by ophthalmic clinicians. We shall use this variable during the external update of the model. We shall collect data on total number of non-attendance and cancellations and no of > 1 consecutive non-attendance or cancellations before the baseline (first HES appointment records).

Appendix 3: Important definitions

- Early worsening: "DR progression to treatment requiring stage during the first year after rapid improvement in blood glucose will be considered EWDR" if there is history of intensive treatment / bariatric / pancreatic surgery (4).
- Follow up: From the first appointment after referral by DESP to first treatment (laser / injection) or vision failure, whichever comes first, death, discharge, transfer or end of the study.
- Outcome: This is a composite of treatment (photocoagulation, injection, vitrectomy) or vision failure (vision loss or blindness)
- Treatment: photocoagulation, Intraocular injection treatment with any anti VEGF or steroid injections laser or vitreous surgery
- Vision failure: Loss of three lines of vision (10 to 15 letters on EDTRS) or more, only if it happens before treatment. (Conversions-Between-Letter-LogMAR-and-Snellen-Visual-Acuity-Scores.png (605×725) (researchgate.net), (VALIDITY OF OUTCOME MEASURES - Ranibizumab (Lucentis) - NCBI Bookshelf (nih.gov)

Supplementary File

For peer review only - http://bmjopen.bmj.com/site/about/guidelines.xhtml

References

1. James Lind Alliance. Priority Setting Partnerships, Retinal Vascular Disease Top 10 [cited 2013. Available from: https://www.jla.nihr.ac.uk/priority-setting-partnerships/sight-loss-and-vision/top-10-priorities/retinal-vascular-disease-top-10.htm.
2. Haider S, Sadiq SN, Lufumpa E, Sihre H, Tallouzi M, Moore DJ, et al. Predictors for diabetic retinopathy progression—findings from nominal group technique and Evidence review. BMJ Open Ophthalmology. 2020;5(1):e000579.
3. Staniszewska S, Brett J, Simera I, Seers K, Mockford C, Goodlad S, et al. GRIPP2 reporting checklists: tools to improve reporting of patient and public involvement in research. BMJ. 2017;358:j3453.
4. Feldman-Billard S, Larger E, Massin P. Early worsening of diabetic retinopathy after rapid improvement of blood glucose control in patients with diabetes. Diabetes Metab. 2018;44(1):4-14.
5. Klein BE, Moss SE, Klein R. Effect of pregnancy on progression of diabetic retinopathy. Diabetes care. 1990;13(1):34-40.
6. DCCT. Effect of pregnancy on microvascular complications in the diabetes control and complications trial. The Diabetes Control and Complications Trial Research Group. Diabetes care. 2000;23(8):1084-91.
7. Rasmussen KL, Laugesen CS, Ringholm L, Vestgaard M, Damm P, Mathiesen ER. Progression of diabetic retinopathy during pregnancy in women with type 2 diabetes. Diabetologia. 2010;53(6):1076-83.
8. Forster AS, Forbes A, Dodhia H, Connor C, Du Chemin A, Sivaprasad S, et al. Non-attendance at diabetic eye screening and risk of sight-threatening diabetic retinopathy: a population-based cohort study. Diabetologia. 2013;56(10):2187-93.

For peer review only - http://bmjopen.bmj.com/site/about/guidelines.xhtml
