# Supplementary material for: Diabetic retinopathy progression in patients under monitoring for treatment or vision loss: external validation and update of a multivariable prediction model
Source: BMJ Open. 2023 Apr 3;13(4):e073015. doi: 10.1136/bmjopen-2023-073015 (PMC10083856; doi:10.1136/bmjopen-2023-073015)
Supplement: Reviewer comments [file bmjopen-2023-073015.reviewer_comments.pdf]

PEER REVIEW HISTORY

BMJ Open publishes all reviews undertaken for accepted manuscripts. Reviewers are asked to complete a checklist review form (**http://bmjopen.bmj.com/site/about/resources/checklist.pdf**) and are provided with free text boxes to elaborate on their assessment. These free text comments are reproduced below.

ARTICLE DETAILS

**TITLE (PROVISIONAL)**	Diabetic Retinopathy Progression in patients under monitoring for treatment or vision loss: External Validation, and update of a Multivariable Prediction Model.
**AUTHORS**	Haider, Sajjad; Adderley, Nicola; Tallouzi, Mohammad O.; Sadiq, Salman; Steel, David; Chavan, Randhir; Sheikh, Ijaz; Nirantharakumar, Krishnarajah; Snell, Kym IE

This article was not externally reviewed at BMJ Open. Protocols that have been independently assessed prior to submission to BMJ Open are usually fast-tracked to publication on the grounds that further substantial changes will not be possible. This independent assessment will usually be external, independent review for both a substantial grant award from a non-commercial or government funder and ethics approval.
